# The *Fox* Gene Repertoire in the Annelid *Owenia fusiformis* Reveals Multiple Expansions of the *foxQ2* Class in Spiralia

**DOI:** 10.1093/gbe/evac139

**Published:** 2022-09-13

**Authors:** Océane Seudre, Francisco M Martín-Zamora, Valentina Rapisarda, Imran Luqman, Allan M Carrillo-Baltodano, José M Martín-Durán

**Affiliations:** School of Biological and Behavioural Sciences, Queen Mary University of London, Mile End Road, E1 4NSUnited Kingdom; School of Biological and Behavioural Sciences, Queen Mary University of London, Mile End Road, E1 4NSUnited Kingdom; School of Biological and Behavioural Sciences, Queen Mary University of London, Mile End Road, E1 4NSUnited Kingdom; School of Biological and Behavioural Sciences, Queen Mary University of London, Mile End Road, E1 4NSUnited Kingdom; School of Biological and Behavioural Sciences, Queen Mary University of London, Mile End Road, E1 4NSUnited Kingdom; School of Biological and Behavioural Sciences, Queen Mary University of London, Mile End Road, E1 4NSUnited Kingdom

**Keywords:** *Forkhead* genes, gene family evolution, phylogenetics, gene expression, *Owenia fusiformis*, sub-functionalization

## Abstract

*Fox* genes are a large and conserved family of transcription factors involved in many key biological processes, including embryogenesis and body patterning. Although the role of *Fox* genes has been studied in an array of model systems, comprehensive comparative studies in Spiralia—a large clade of invertebrate animals including molluscs and annelids—are scarce but much needed to better understand the evolutionary history of this gene family. Here, we reconstruct and functionally characterize the *Fox* gene complement in the annelid *Owenia fusiformis*, a slow evolving species and member of the sister group to all remaining annelids. The genome of *O. fusiformis* contains at least a single ortholog for 20 of the 22 *Fox* gene classes that are ancestral to Bilateria, including an ortholog of the recently discovered *foxT* class. Temporal and spatial expression dynamics reveal a conserved role of *Fox* genes in gut formation, mesoderm patterning, and apical organ and cilia formation in Annelida and Spiralia. Moreover, we uncover an ancestral expansion of *foxQ2* genes in Spiralia, represented by 11 paralogs in *O. fusiformis*. Notably, although all *foxQ2* copies have apical expression in *O. fusiformis,* they show variable spatial domains and staggered temporal activation, which suggest cooperation and sub-functionalization among *foxQ2* genes for the development of apical fates in this annelid. Altogether, our study informs the evolution and developmental roles of *Fox* genes in Annelida and Spiralia generally, providing the basis to explore how regulatory changes in *Fox* gene expression might have contributed to developmental and morphological diversification in Spiralia.

**Significance**

The role of *Fox* genes, a group of DNA-binding proteins required for the formation of many animal organs, is poorly understood in invertebrate groups such as molluscs and annelids. Here, by studying the genome and embryogenesis of the annelid *Owenia fusiformis*, we demonstrate that *Fox* genes are involved in the development of the gut, muscles, cilia, and nervous system. Importantly, we find that a group of *Fox* genes (referred to as *foxQ2*) expressed in the anterior end of most animals has more copies in annelids and molluscs than in other invertebrate groups like insects and sea stars. Together, our findings clarify the evolution of *Fox* genes and their contribution to the diversity of forms and organs found in marine invertebrates.

## Introduction

Forkhead box-containing proteins (i.e. *Fox* genes) form one of the largest families of transcription factors in animals, displaying a remarkable functional diversity in many morphogenetic processes (Kaufmann and Knöchel 1996; Carlsson and Mahlapuu 2002; Hannenhalli and Kaestner 2009). *Fox* genes are characterized by a conserved DNA-binding domain of approximately 100 amino acids—the Forkhead or winged helix domain—that folds into two stereotypical large loops or “wings” (Clark *et al*. 1993; Li and Tucker 1993). Since the discovery of the Forkhead domain in the fruit fly *Drosophila melanogaster* (Weigel *et al*. 1989), *Fox* genes have been studied in a wide range of traditional developmental systems, mostly vertebrates (Mazet *et al*. 2003; Lee and Frasch 2004; Hannenhalli and Kaestner 2009; Jackson *et al*. 2010; Golson and Kaestner 2016). The initial description of 15 *Fox* gene classes in chordates, each identified by a letter (Kaestner *et al*. 2000), and the establishment of a unified nomenclature facilitated phylogenetic analyses and comparisons with other major invertebrate clades, such as hemichordates (Fritzenwanker *et al*. 2014), molluscs (Yang *et al.*, 2014; Wu *et al.*, 2020), platyhelminthes (Pascual-Carreras *et al*. 2021), panarthropods (Schomburg *et al*. 2022), cnidarians (Magie *et al*. 2005), and sponges (Larroux *et al*. 2006), as well as animal outgroups (Larroux *et al*. 2008). This uncovered a complex evolutionary history for this large family of transcription factors. Today, *Fox* genes are classified into as many as 27 classes belonging to two major clades (Kaestner *et al*. 2000; Mazet *et al*. 2003; Larroux *et al*. 2008; Hannenhalli and Kaestner 2009; Benayoun *et al*. 2011; Pascual-Carreras *et al*. 2021; Schomburg *et al*. 2022), where ancestral duplication events (e.g. the former class *foxQ* split into *foxQ1* and *foxQ2*, *foxN* into *foxN1/4* and *foxN2/3*, *foxL* into *foxL1* and *foxL2/3*, and *foxJ* into *foxJ1* and *foxJ2/3*), gene innovations (e.g. *foxR* and *foxS* are unique of vertebrates, *foxT* is potentially a novelty of panarthropods), expansions and losses (e.g. *foxAB* in vertebrates, *foxQ2* in tetrapods, and *foxAB*, *foxE*, and *foxI* in panarthropods) are common (Mazet *et al*. 2003; Tu *et al*. 2006; Wotton and Shimeld 2006; Paps *et al*. 2012; Schomburg *et al*. 2022). Moreover, genomic comparisons also uncovered signs of conserved syntenic linkage for some of the classes, such as *foxL1*-*foxC*-*foxF*-*foxQ1*, in phylogenetically distant lineages of insects, chordates and spiralians (Mazet *et al*. 2006; Wotton and Shimeld 2006, 2011; Wotton *et al*. 2008; Shimeld *et al.* 2010a; Schomburg *et al*. 2022). Yet a comprehensive characterization of *Fox* genes is still lacking in most major animal groups, most notably in members of Spiralia, one of the two main clades of protostomian animals that comprises nearly half of the extant major metazoan groups, including molluscs and annelids (Marlétaz *et al*. 2019). Therefore, the evolutionary history and developmental roles of this conserved family of transcription factors are still unclear at key nodes of the animal tree of life.

*Fox* genes typically show tissue-specific expression patterns and play an important role in cell-type determination and differentiation (Hannenhalli and Kaestner 2009; Jackson *et al*. 2010). Functional studies in human, mouse, zebrafish, and fly have revealed an array of functions of *Fox* genes in early development, such as axial patterning, germ layer specification, and organogenesis (reviewed in Carlsson and Mahlapuu, 2002). In Spiralia, however, studies on the function of *Fox* genes are scarce and mostly focused on certain classes, with just a handful of studies encompassing more than one major spiralian clade (Supplementary table 1 and references therein, Supplementary material online). For example, *foxA* is consistently expressed in the developing foregut in many spiralians, including annelids, brachiopods, phoronids, and bryozoans (Arenas-Mena 2006; Boyle and Seaver 2008, 2010; Adler *et al*. 2014; Martín-Durán *et al*. 2016; Vellutini *et al*. 2017; Kwak *et al*. 2018; Andrikou *et al*. 2019; Kostyuchenko *et al*. 2019) and *foxJ1*, *foxQ2* and *foxG* are expressed in larval specific tissues in the annelid *Platynereis dumerilii*, the brachiopod *Terebratalia transversa* and the phoronid *Phoronopsis harmerii* (Santagata *et al*. 2012; Marlow *et al*. 2014; Gąsiorowski and Hejnol 2020). Similarly, the clustered classes *foxC*, *foxL1* and *foxF* show mesodermal expression in all spiralian species studied to date, suggesting that coordinated activation of these *Fox* genes in a common germ layer might have contributed to the maintenance of their genetic linkage (Shimeld *et al.* 2010a; Passamaneck *et al*. 2015; Martín-Durán *et al*. 2016). Other *Fox* gene classes, however, have only been studied in individual species, which prevents inferring an ancestral role for these genes in Spiralia. For example, *foxL2/3* is a regulator of ovarian differentiation and development in molluscs (Liu *et al*. 2012; Yang *et al*. 2014; Teaniniuraitemoana *et al*. 2015; Li *et al*. 2016), *foxB* is expressed during late mesoderm development in the leech *Helobdella austinensis* (Kwak *et al*. 2018), *foxO* controls tissue regeneration and cell death in the planarian *Schmidtea mediterranea* (Pascual-Carreras *et al*. 2021) and *foxK* is involved in ectodermal regeneration in that same planarian species (Coronel-córdoba *et al*. 2022). Consequently, the repertoire and developmental functions of most *Fox* genes remain largely unexplored in Spiralia, and thus its study is not only important to discern the evolution of this gene family in animals, but also the contribution of these developmental regulators to the diversification of body plans and embryonic modes in this major animal group.

Here, we mined the genome of the annelid *Owenia fusiformis* (Martín-Zamora *et al.* 2022), a member of Oweniidae and sister group to all remaining annelids (Rouse *et al*. 2022), and eight other annelids with high-quality genomic and transcriptomic datasets to infer the ancestral *Fox* gene complement to Annelida, one of the most species-rich and morphologically diverse groups within Spiralia. Temporal and spatial gene expression analyses offer insights into the potential role of some of the *Fox* gene classes in *O. fusiformis*, uncovering conserved and putative new roles for some of the *Fox* classes. Moreover, our study reveals that the *foxQ2* class is largely expanded in Spiralia, with the paralogs being consistently expressed in apical territories and exhibiting signs of possible sub-functionalization in *O. fusiformis*. Altogether, our study informs the evolution of the *Fox* gene family in Annelida, providing valuable data to reconstruct the evolution and developmental roles of these genes in Spiralia and Metazoa.

## Results

### The *Fox* Gene Complement in *O. fusiformis*

**Fig. 1.**
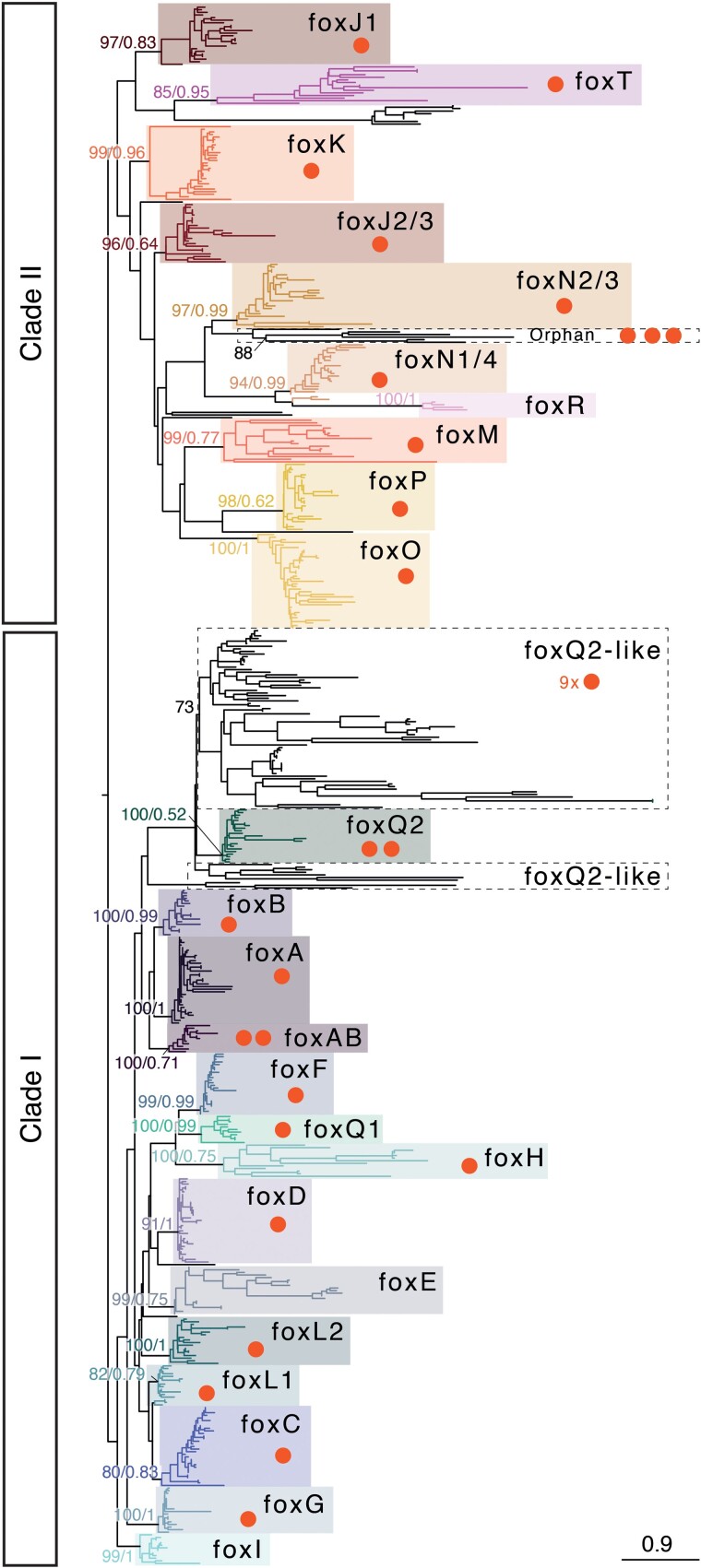
Gene orthology analysis of *Fox* genes. Orthology assignment of *Fox* genes in all major *Fox* classes in *O. fusiformis* and eight other annelid species (*D. gyrociliatus*, *P. dumerilii*, *C. teleta*, *L. luymesi*, *P. echinospica*, *S. benedicti*, *E. andreii*, *H. robusta*). The tree topology is based on maximum likelihood reconstruction and node supports indicate both bootstrap values (from 0 to 100) and posterior probabilities (from 0 to 1) at key nodes. Boxes indicate each *Fox* gene class and dots indicate the presence and number of copies in *O. fusiformis* genome. Scale bars indicate the number of amino acids substitutions per site alongside the branches. See Supplementary figs. 2 and 3, Supplementary material online for the fully annotated trees.

**Fig. 2.**
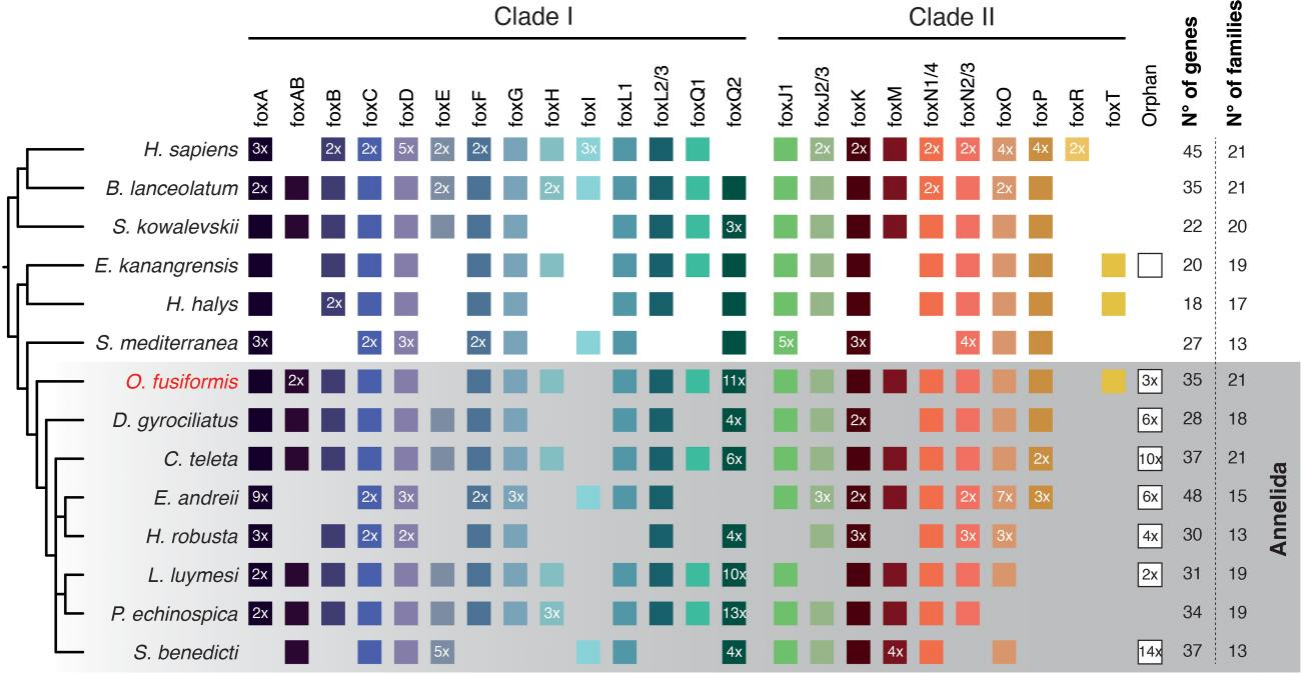
The *Fox* gene complement in *O. fusiformis* and selected metazoan clades. Schematic summary of the *Fox* gene repertoires in *O. fusiformis* and seven annelid species with high-quality, publicly available genomes, in comparison with selected bilaterian taxa belonging to Spiralia (*S. mediterranea*), Ecdysozoa (*E. kanangrensis* and *H. halys*) and Deuterostomia (*S. kowalevskii*, *B. lanceolatum*, *H. sapiens*). Boxes indicate the presence of an ortholog and numbers inside specify the number of paralogs per class and species. The presence of *foxT* in *C. teleta* is only hinted by maximum likelihood analyses.

To characterize the *Fox* gene complement in the annelid *O. fusiformis,* we searched for annotated gene models containing the conserved Forkhead DNA-binding domain in its reference genome (Martín-Zamora *et al.* 2022) and in eight other species with high quality, publicly available genomic and transcriptomic datasets (Simakov *et al*. 2013; Chou *et al*. 2018; Li *et al*. 2019; Shao *et al*. 2020; Martín-Durán *et al*. 2021; Sun *et al*. 2021; Zakas *et al*. 2022). We obtained a total of 35 putative *Fox* genes in *O. fusiformis*, and between 28 and 48 in other annelids, which is a higher number than those previously reported in other Spiralian species, in which the number of *Fox* genes ranges from 21 to 26 genes (Yang *et al.* 2014; Wu *et al*. 2020; Pascual-Carreras *et al*. 2021). To assign the orthology of each of the *O. fusiformis Fox* genes, we applied maximum likelihood and Bayesian phylogenetic tree inference, obtaining strongly supported orthologs for 21 of the 24 classes of *Fox* genes, both in clade I and clade II (figs. 1 and 2; Supplementary figs. 1–3, Supplementary material online). Only the two ancestral bilaterian *Fox* gene classes *foxE* an *foxI* are missing in *O. fusiformis*, which have been however reported in other spiralian lineages (Yang *et al.* 2014; Wu *et al*. 2020). Nonetheless, these two *Fox* gene classes show a dynamic pattern of loss/expansion in Annelida, with *foxE* being lost in earthworms and leeches and expanded in the spionid *Streblospio benedicti*, and *foxI* being generally lost in Annelida and only retained in the earthworm *Eisenia andrei* and *S. benedicti* (fig. 2). Indeed, earthworms, leeches, and *S. benedicti* have divergent *Fox* gene complements, with many losses and expansions, in contrast to most other annelid species that have relatively stable and more complete *Fox* gene repertoires (fig. 2; Supplementary figs. 2 and 3, Supplementary material online). Notably, *O. fusiformis* is the only annelid to have a *bona fide foxT* ortholog (Supplementary figs. 2–4, Supplementary material online), which indicates that this recently described *Fox* gene class in Panarthropoda is more ancient that initially thought (Schomburg *et al*. 2022). In addition, *O. fusiformis* has 14 fast-evolving orthologs that are either related to *foxQ2* in clade I or *foxN2/3* in clade II (fig. 1; Supplementary figs. 2 and 3, Supplementary material online). While the 11 *foxQ2*-like genes could be confidently assigned to the *foxQ2* class (see below), the orthology of the divergent *Fox* genes belonging to clade II could not be resolved and thus we deem them “orphan” genes (figs. 1 and 2). Similarly, most other annelids show a varying number of orphan *Fox* genes (fig. 2), most of them related to *foxQ2* and *foxN2/3* classes (Supplementary figs. 2 and 3, Supplementary material online), albeit *Capitella teleta* has a large expansion of eight *Fox* genes that show certain similarity with the *foxT* class under maximum likelihood orthology assignment (Supplementary fig. 2, Supplementary material online). Together, our findings reconstruct the *Fox* gene complement for *O. fusiformis* and other annelids (fig. 1), thus helping to infer an ancestral *Fox* repertoire to this major animal group and Spiralia in general.

### Genomic Architecture and Chromosomal Linkage of *Fox* Genes in *O. Fusiformis*

**Fig. 3.**
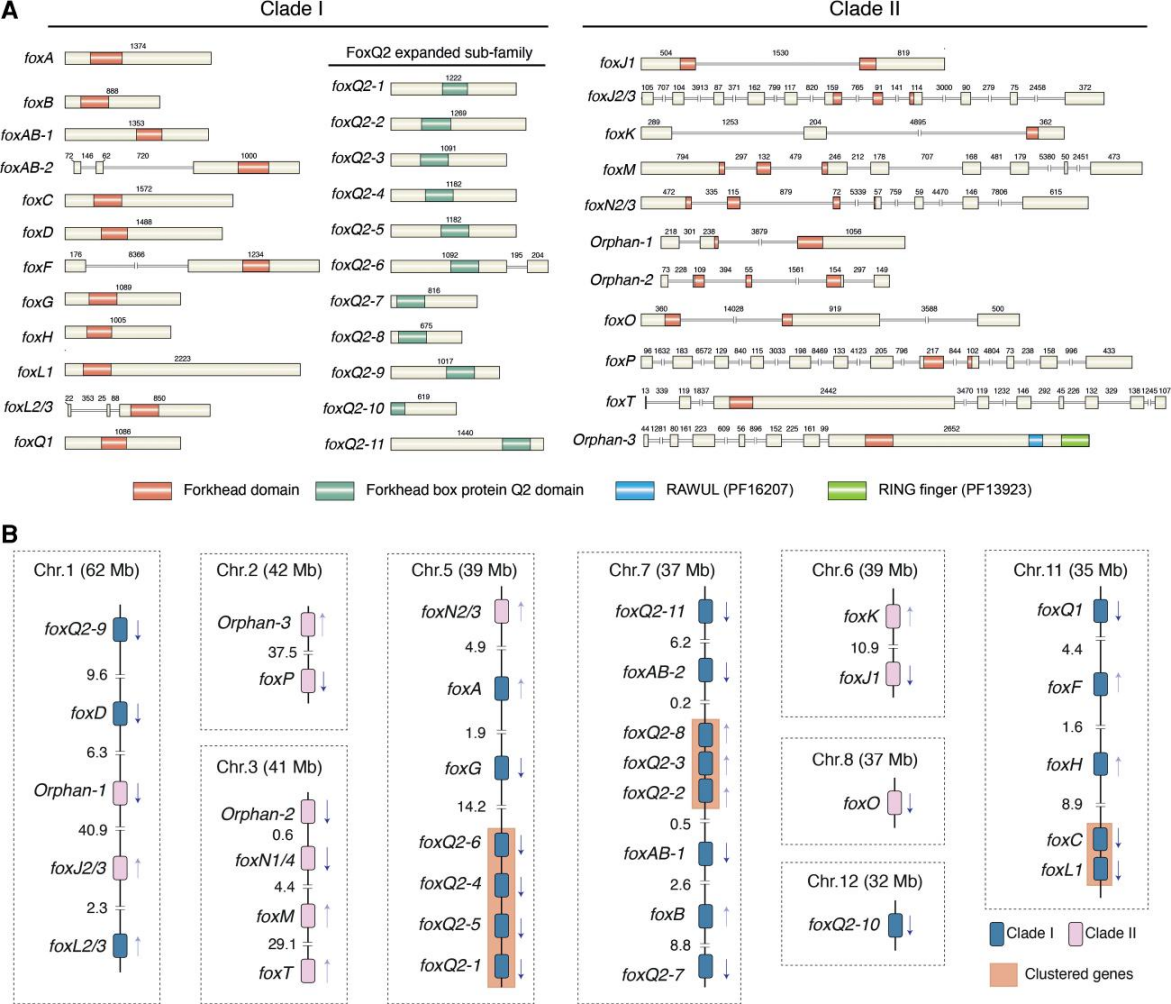
The genomic architecture and linkage of *Fox* genes in *O. fusiformis*. (*A*) Schematic representation of the protein domain composition and exon-intron structure for the *Fox* genes in *O. fusiformis*. Boxes indicate coding regions and double black lines represent introns (double oblique lines mean the scale is not proportional and number above exons and introns indicate their length in base pairs), with the locations of the Forkhead domains (PF00250), the FoxQ2 specific domain (CD20035), and the additional protein domains present in the *Fox* orphan-3 gene indicated by internal differentially shaded areas. The *foxK* and *foxQ2-10* gene models are truncated in the N-terminus. The genomic structure for the manually curated *foxN1/4* gene could not be resolved. (*B*) Schematic drawings of the genomic organization of the *Fox* genes in *O. fusiformis*. For each chromosome (Chr; chromosome size in brackets and in Mb), numbers between genes indicate major intergenic distances in Mb and arrows indicate the transcriptional orientation of each gene. *Fox* genes belonging to Clade I and Clade II are in different tones. Clustered genes are highlighted with shaded boxes.

*Fox* genes belonging to clade I and clade II additionally differ in whether they lack or contain introns at conserved sites of the Forkhead domain, respectively (Larroux *et al*. 2008). To further characterize the *Fox* gene complement and assess this rule in *O. fusiformis*, we reconstructed the domain architecture and exon-intron composition of all 35 *Fox* genes in this annelid. *Fox* genes in *O. fusiformis* show diverse gene architectures, with lengths ranging from 675 bp (*foxQ2-8*) to 3,372 bp (*fox* orphan-3), and all but *foxQ2-10* contain an intact Forkhead domain (fig. 3*A*). One *Fox* gene—*fox* orphan-3—shows two additional protein domains, namely a RING finger and RAWUL domains (fig. 3*A*), which we confirmed by RNA-seq sequencing, and that might indicate that this divergent *Fox* gene have acquired new protein functions. Exon numbers in the *Fox* genes of *O. fusiformis* range from one to 11, and most genes follow the clade I and clade II distinction based on the number of introns, apart from *foxAB-2, foxF*, *foxL2/3*, and *foxQ2-6*, which contain introns albeit they belong to clade I and might thus represent independent intron gains (fig. 3*A*; Supplementary table 2, Supplementary material online). Altogether, these findings reinforce the previous observation that *O. fusiformis* contains a relatively well conserved *Fox* gene repertoire, while they highlight as well that a handful of *Fox* gene classes and paralogs might have experience faster rates of molecular evolution.

Certain *Fox* gene classes (e.g. *foxL1*, *foxC*, *foxF*, and *foxQ1*) show conserved chromosomal linkage across phylogenetically distant bilaterian taxa, such as panarthropods, vertebrates and amphioxus, and there are evidences of this linkage in some spiralian species (Mazet *et al*. 2006; Shimeld *et al.* 2010a; Shimeld *et al.* 2010b; Schomburg *et al*. 2022). To assess whether this feature was also retained in *O. fusiformis*, we studied the chromosomal location and microsyntenic relationship of the 35 *Fox* genes in this annelid. *Fox* genes are spread across nine of the 12 chromosomes of *O. fusiformis*, namely chromosomes 1, 2, 3, 5, 6, 7, 8, 11, and 12 (fig. 3*B*). While *foxF*, *foxC*, *foxL1*, and *foxQ1* are located on the same chromosome—chromosome 11—only *foxC* and *foxL1* show evidence of a tight linkage, with a high number of genes (>1,000) lying between f*oxQ1* and *foxF* as well as between *foxF* and *foxC* (fig. 3*B*). In addition, we observed tandem duplications of *foxQ2* genes in chromosomes 5 and 7 (fig. 2*B*). Therefore, even though the ancestral bilaterian chromosomal linkage is overall conserved in *O. fusiformis* (Martín-Zamora *et al.* 2022), the ancestral microsyntenic relationships observed among certain *Fox* genes is lost in this annelid species.

### The Expression Dynamics of the *Fox* Gene Complement in *O. Fusiformis*

**Fig. 4.**
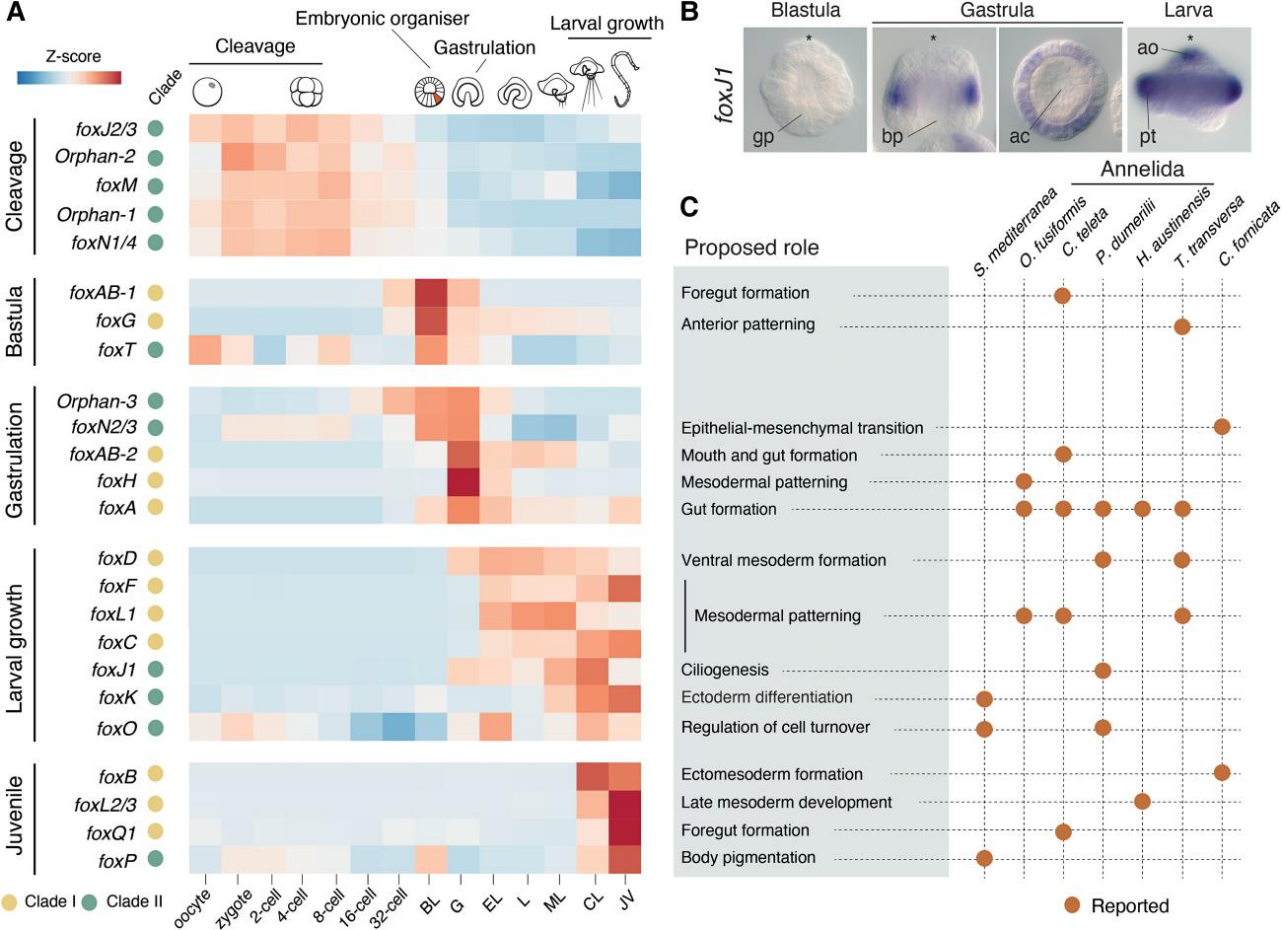
Temporal dynamics of expression of the *Fox* genes in *O. fusiformis* and their role in Spiralia. (*A*) Heatmap of expression of all *Fox* genes (except for the *foxQ2* family, which is shown in fig. 5) throughout development in *O. fusiformis*, as normalized z-score expression values. The x-axis shows the developmental time points (BL: Blastula; G: Gastrula; EL: Elongation; L: Larva; ML: Mitraria larva; CL: Competent Larva; and JV: Juvenile). The second column from the left with dots indicates the membership of each *Fox* gene to either Clade I or Clade II. Genes are ordered vertically following their timing of expression: maternal and during early cleavage; at the time of specification of the organizer cell (5 hours post-fertilization; highlighted with a coloured area in the schematic drawing); during gastrulation; during larval growth; and at the juvenile stage. (*B*) Whole mount *in situ* hybridization of *foxJ1* gene during the development of *O. fusiformis*, at the blastula (lateral view), gastrula (lateral to the left and ventral to the right) and larval stages (lateral view). Consistent with the temporal expression data, *foxJ1* starts to be expressed at the putative prototroch precursor cells at the gastrula stage and it is later detected in the ciliated cells of the larva (apical organ and prototroch). Asterisks mark the apical/anterior pole. ac, archenteron; ao, apical organ; bp, blastopore; gp, gastral plate; pt, prototroch. (*C*) Table summarizing the current knowledge of the developmental roles of *Fox* genes in Spiralia (from Supplementary table 1). For each gene, reported developmental roles are in the light coloured box to the left and the associated species are shown with a dot on the corresponding horizontal line on the right.

To investigate the expression dynamics of the *Fox* genes in *O. fusiformis* and relate each of these genes to major morphogenetic events during the life cycle of this annelid, we used available stage-specific RNA-seq data covering 14 developmental time points, from the unfertilized oocyte to the juvenile stage (Martín-Zamora *et al.* 2022). In *O. fusiformis*, the temporal expression dynamics of the *Fox* genes seem to correlate with their assignment to clade I and clade II, because half (6) of the clade II *Fox* genes are expressed maternally and during the early cleavage stages, while clade I *Fox* genes tend to show short peaks of expression at single developmental stages, from the 32-cell stage onwards (fig. 4*A*). Clade II genes that escape this trend are *foxP*, expressed at the juvenile stage (but also briefly at the blastula stage), *foxN2/3* and the orphan-3 gene, which peak during gastrulation, and *foxJ1*, *foxK*, and *foxO*, that are expressed during larval development (fig. 4*A*). Notably, a set of *Fox* genes belonging to clade I (*foxAB-1*, *foxG*, and many *foxQ2* paralogs, see below) are finely expressed at the time of the specification of the embryonic organizer and the establishment of the axial identities in *O. fusiformis*, as well as during gastrulation (i.e. *foxAB-2, foxH, foxA*) (Carrillo-Baltodano *et al*. 2021; Seudre *et al*. 2022) (fig. 4*A*). Other genes from the same clade become expressed later during embryogenesis and are probably associated with either organogenesis and larval development (i.e. *foxD*, *foxF*, *foxL1*, *foxC*) or juvenile metamorphosis (i.e. *foxB, foxL2/3, foxQ1*) (fig. 4*A* and *C*). We confirmed these temporal expression profiles for six *Fox* genes, namely *foxJ1*, *foxK*, *foxM*, *foxN1/4*, *foxN2/3* and *foxP* (Supplementary fig. 5, Supplementary material online). While the gene *foxJ1*, which is a conserved regulator of cilia development (Xianwen Yu et al. 2008), is expressed in the cells forming the apical tuft and ciliated band in the larva of *O. fusiformis* (fig. 4*B*), *foxK* is expressed in the anterior and ventral ectoderm, *foxN1/4* and *foxP* in the apical ectoderm of the blastula, *foxN2/3* in the endoderm during gastrulation, and *foxM* broadly in the blastula (Supplementary fig. 5, Supplementary material online). Together, these data support an embryonic role for most *Fox* genes in *O. fusiformis*, revealing diverse expression dynamics that correlate with crucial cell-type specification and morphogenetic events.

### *O. fusiformis* has an Expanded *foxQ2 C*lass

**Fig. 5.**
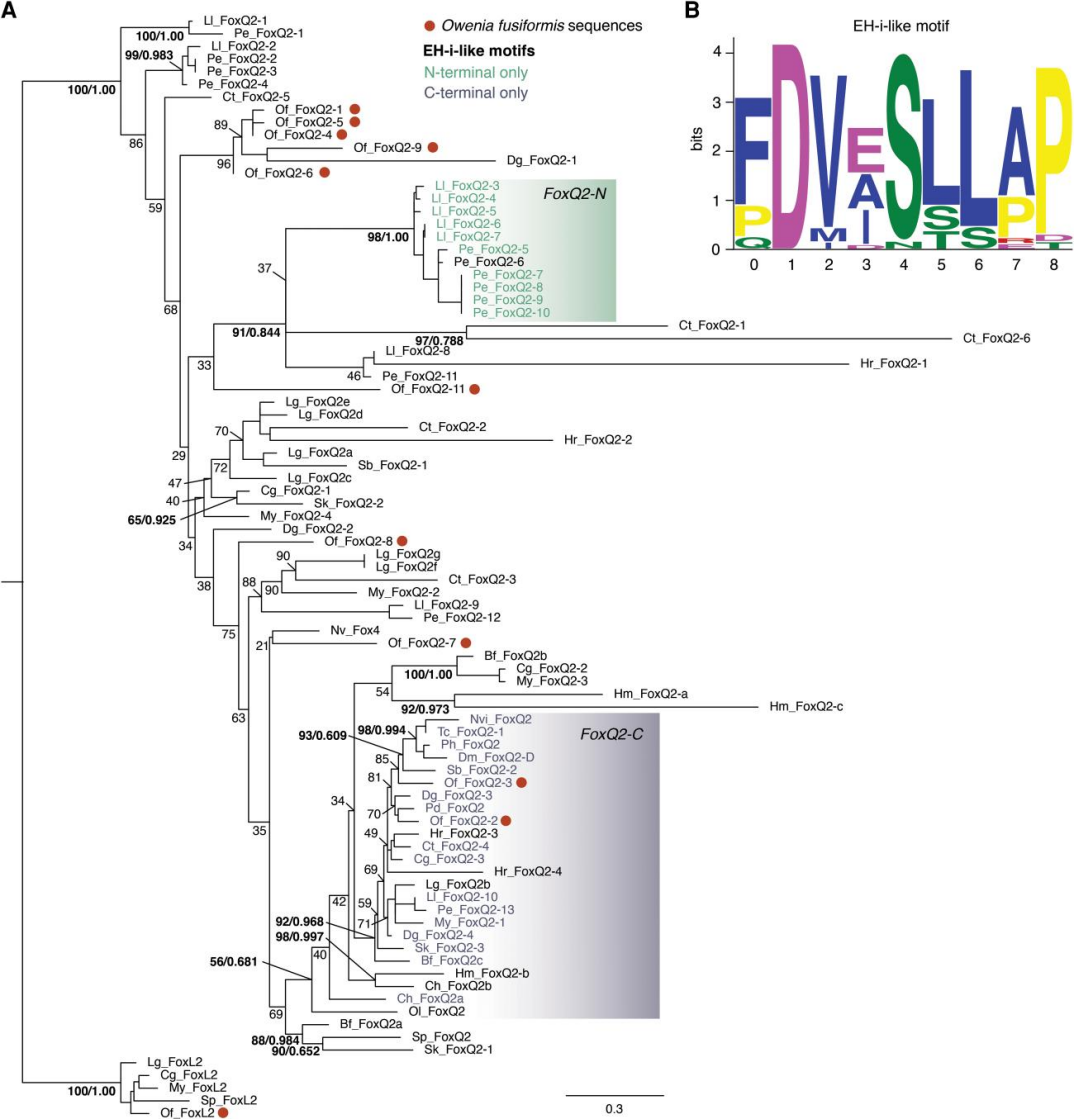
Phylogenetic analysis of the *foxQ2* family. (*A*) Maximum likelihood tree topology of the *foxQ2* class with *foxL2* class as outgroup. Sequence names are colored based on the position of the EH-i-like motif (shown in [*B*]) with respect to the FoxQ2 box. The coloured box at the bottom of the tree highlights the monophyletic clade largely containing sequences of both cnidarian and bilaterian lineages with a C-terminal EH-i-like motif. Dots indicate *O. fusiformis foxQ2* paralogs. Only sequences with a fully intact FoxQ2 domain (accession number CD20035) are included in the analysis (e.g. *foxQ2-10* from *O. fusiformis* is not included). (*B*) Sequence logo of the EH-i-like motif we identified.

Previous studies indicated that an ancestral duplication in the *foxQ2* class occurred at least in the last common bilaterian ancestor, and maybe even predated the cnidarian-bilaterian split (Chevalier *et al*. 2006; Fritzenwanker *et al*. 2014; Pascual-Carreras *et al*. 2021), which was followed by further duplications of this *Fox* gene class in some spiralian and deuterostomian lineages (Yang *et al.* 2014; Wu *et al*. 2020). This observation agrees with the large expansion of 11 *foxQ2* paralogs that we identified in the annelid *O. fusiformis* (fig. 1), which is mirrored in many other annelid lineages. Moreover, the presence of an Engrailed Homology 1 (EH)-i-like Groucho binding motif in some *foxQ2* orthologs and its variable C- and N-terminal position with respect to the Forkhead and FoxQ2 domain has been used to subdivide the *foxQ2* class in *foxQ2-C* and *foxQ2-N*, or even to define a new sub-class named *foxQD* (Fritzenwanker *et al*. 2014; Pascual-Carreras *et al*. 2021). To clarify the evolution of this *Fox* gene class, and how the expansions of *foxQ2* genes occurred in *O. fusiformis* and spiralians generally, we mined available databases and the genomes of seven annelid species in search for genes with complete FoxQ2 domains, which we then used for phylogenetic reconstruction and the identification of EH-i-like motifs. This is a stringent approach that supported the ascription of most of the divergent clade I *Fox* genes to the *foxQ2* class (e.g. the 11 *foxQ2*-like genes of *O. fusiformis*; Supplementary fig. 2, Supplementary material online), with those not meeting the criteria being considered as orphan *Fox* genes (fig. 2). While the general orthology of all identified *foxQ2* genes was robustly supported (fig. 5*A*; Supplementary figs. 6–8, Supplementary material online), we did not recover two separate monophyletic clades with EH-i-like motifs in either the C- or the N-terminus. Instead, *foxQ2* orthologs with an EH-i-like motif at the C-terminal end (fig. 5*B*) appear to form a relatively well supported monophyletic clade, comprising sequences of both cnidarians and most bilaterian groups, often as single copy genes (yet the annelids *O. fusiformis*, *Dimorphilus gyrociliatus*, and *Helobdella robusta* have two paralogs). The rest of the *foxQ2* sequences lack an EH-i-like motif and probably represent more or less divergent copies. Among these fast-evolving *foxQ2* copies we found lineage-restricted expansions, such as those of *O. fusiformis*—for which the phylogenetic relationship between paralogs correlate well with their genomic linkage—and the vestimentiferan annelids *Lamellibrachia luymesi* and *Paraescarpia echinospica*, which have a group of *foxQ2* genes that independently aquired an EH-i-like motif on the N-terminal end (fig. 5*A*). Together, our findings corroborate previous analyses revealing a complex evolutionary history for the *foxQ2* gene class, probably dominated by fast rates of molecular evolution and/or frequent independent events of gene duplication in both cnidarian and bilaterian lineages, specially among spiralians, as well as its complete loss in tetrapods.

### The Developmental Expression of *foxQ2 P*aralogs in *O. fusiformis*

**Fig. 6.**
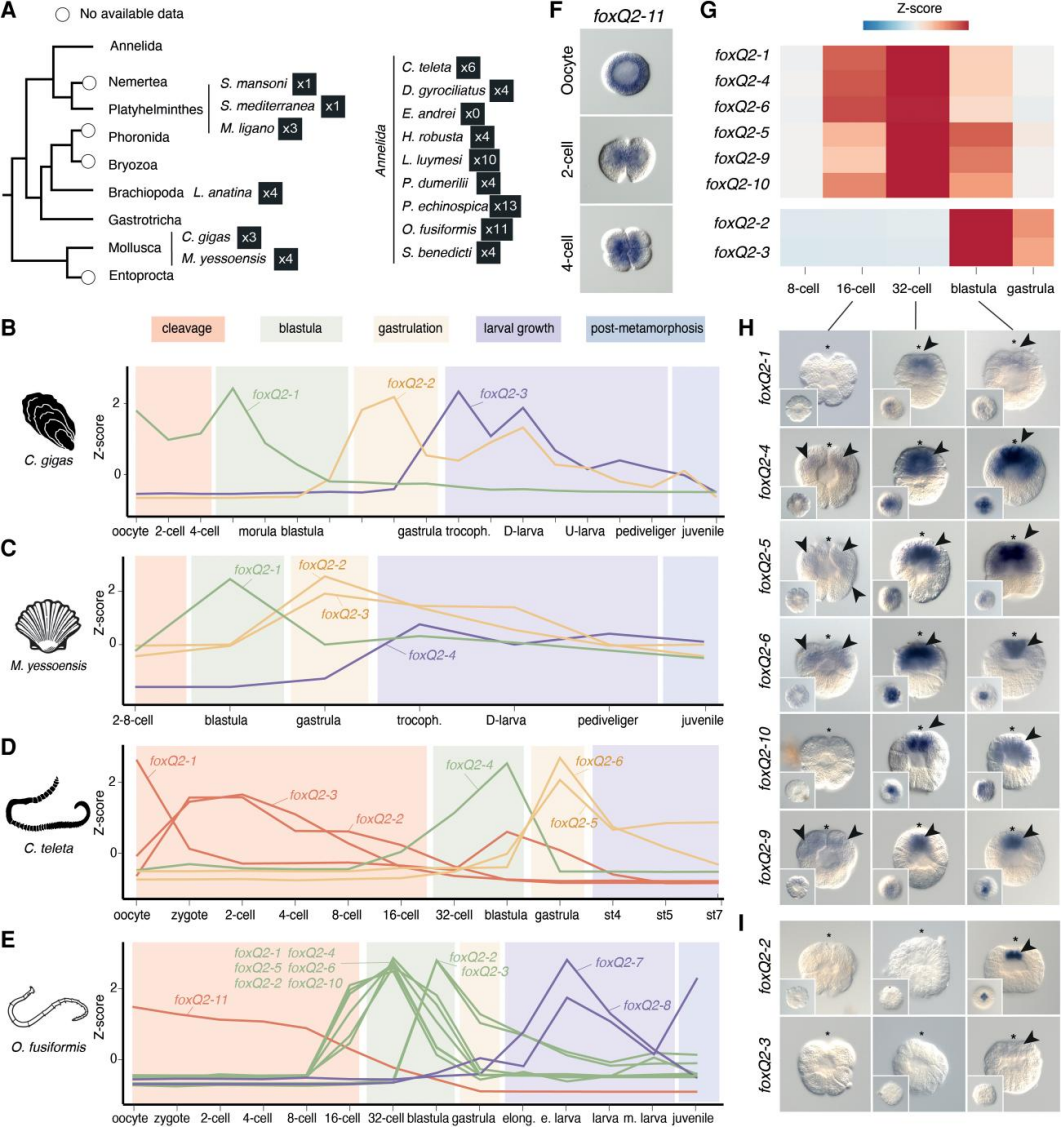
The *foxQ2* gene complement in Annelida and Mollusca. (*A*) Number of *foxQ2* genes in selected spiralian taxa based on the orthology assignment in fig. 5*A*, under a recent phylogenetic framework (Marlétaz *et al*. 2019). (*B–E*) Line plots representing z-score values of the temporal expression dynamics of *foxQ2* paralogs during the development of the molluscs *C. gigas* and *M. yessoensis*, and the annelids *C. teleta* and *O. fusiformis*. Exact times of development are detailed in Supplementary Table 3. Major developmental phases are indicated in colored boxes to help compare phases of development across the four species. The line of expression of each gene is colored according to the stage in which it shows maximal expression. (*F*) Apical views of whole mount *in situ* hybridizations of *foxQ2-11* in *O. fusiformis* at the oocyte stage, the two-cell stage, and the four-cell stage. This gene shows maternal expression and equal distribution in early blastomeres. (*G*) Heatmap of expression of *foxQ2* paralogs with peaks of expression at 4 and 5 hours post-fertilization (blastula) in *O. fusiformis*. Colors show the normalized z-score value of expression. (*H* and *I*) Lateral views of whole mount *in situ* hybridizations of *foxQ2* paralogs expressed from 3 to 5 hours post-fertilization (blastula). Insets show apical views, the asterisks point to the animal/apical pole, and arrowheads to the domains of expression.

To assess the potential functional implications of the expansions of *foxQ2* genes in spiralians (fig. 6*A*), we first compared the developmental expression profiles of *foxQ2* genes in *O. fusiformis* and three other spiralian species, namely the molluscs *Crassostrea gigas* and *Mizuhopecten yessoenssis* and the annelid *C. teleta* (fig. 6*B*–*E*). In the pacific oyster *C. gigas*, the three *foxQ2* paralogs (Supplementary table 5, Supplementary material online) show distinct temporal patterns of expression, with *foxQ2-3* peaking at the trochophore stage, *foxQ2-2* showing highest expression during gastrulation, and *foxQ2-1* being expressed maternally and during the early cleavage stages (fig. 6*B*). Similarly, *foxQ2* genes in the scallop *M. yessoensis* (Supplementary table 5, Supplementary material online) display distinct temporal peaks of expression, with *foxQ2-1* having a maximum at the blastula stage, *foxQ2-2* being strongly expressed during gastrulation, and both *foxQ2-3* and *foxQ2-4* peaking at larval stages (fig. 6*C*). The six *foxQ2* paralogs of *C. teleta* display four different temporal patterns of expression: *foxQ2-1* is expressed in the oocyte*, foxQ2-2* and *foxQ2-3* peak during early cleavage, *foxQ2-4* is expressed at the blastula stage, and *foxQ2-5* and *foxQ2-6* show higher expression values during gastrulation (fig. 6*D*). Consistently, *foxQ2* paralogs are also expressed at distinct developmental stages in *O. fusiformis*, with *foxQ2-11* expressed first maternally and during early cleavage, *foxQ2-1, foxQ2-4, foxQ2-5, foxQ2-6, foxQ2-9, foxQ2-10* showing a peak of expression at the early blastula stage, *foxQ2-2* and *foxQ2-3* exclusively expressed at the time of the specification of the embryonic organizer at 5 hours post-fertilization and their expression being under control of the ERK1/2 signaling pathway (Seudre *et al*. 2022), and finally both *foxQ2-7* and *foxQ2-8* coming up at larval stages (fig. 6*E*). Notably, spiralian *foxQ2* orthologs peaking at the blastula stage (i.e. *foxQ2-2* and *foxQ2-3* in *O. fusiformis*, *foxQ2-4* in *C. teleta*, *foxQ2-1* in *M. yessoensis*, and *foxQ2-2* in *C. gigas*) belong to the strongly supported clade of *foxQ2* genes with a conserved C-terminal EH-i-like motif. Similarly, *foxQ2-1* in *C. teleta* and *foxQ2-11* in *O. fusiformis* are phylogenetically related and both show maternal expression. Therefore, these findings support that some of the expansions of *foxQ2* genes are shared across spiralian lineages and that the expansion of this class of *Fox* genes may also resulted in the evolution of novel expression dynamics.

We next analyzed whether *foxQ2* paralogs also showed varying spatial expression domains in *O. fusiformis* using whole mount *in situ* hybridization across targeted developmental stages. Except for *foxQ2-8* and *foxQ2-7*, for which we did not observe any clear expression pattern at the larval stage, all other nine *foxQ2* paralogs showed distinct domains of expression. The paralog *foxQ2-11*, which has a high expression in the oocyte, is detected in the zygote and appears equally distributed in all blastomeres at the two-cell and four-cell stages (fig. 6*F*). The genes *foxQ2-1, foxQ2-4, foxQ2-5*, *foxQ2-6*, *foxQ2-9*, *foxQ2-10*, which peak at 4 hours post-fertilization (fig. 6*G*) and are probably independent expansions in *O. fusiformis* (fig. 5*A*), are expressed at the apical pole, but encompassing varying areas of expression, from ones restricted to just the animal micromeres (e.g. *foxQ2-6* and *foxQ2-9*) to nearly the entire animal hemisphere (e.g. *foxQ2-4*, *foxQ2-5* and *foxQ2-6*) (fig. 6*H*). Finally, the expression of the two *foxQ2* paralogs with a C-terminal EH-i-like motif—*foxQ2-2* and *foxQ2-3*—is restricted to the apical-most micromeres from 5 hours post-fertilization onwards, consistent with the observed temporal dynamics and its regulation by ERK1/2 activity at that timepoint (fig. 6*G* and *I*). Altogether, our gene expression analyses support that the multiple copies of *foxQ2* have retained the evolutionarily conserved expression of this *Fox* gene class in anterior/apical development (Yaguchi *et al*. 2008; Santagata *et al*. 2012; Hunnekuhl and Akam 2014; Marlow *et al*. 2014; Fritzenwanker *et al*. 2014; Martín-Durán *et al*. 2015; Janssen 2017; He *et al*. 2019; Schacht *et al*. 2020), yet they have probably undergone temporal, spatial, and regulative sub-functionalization.

## Discussion

### The Evolution of the *Fox* Gene Complement in Annelida and Spiralia

Taking advantage of the reference genome assembly and comprehensive functional data available for the annelid *O. fusiformis* (Martín-Zamora *et al.* 2022), our study identified and characterized the full *Fox* gene repertoire in a member of Oweniidae, the sister lineage to all remaining annelids (Rouse *et al*. 2022). The 35 *Fox* genes of *O. fusiformis*, belonging to 20 of the 22 classes predicted for the bilaterian ancestor (Kaestner *et al*. 2000; Benayoun *et al*. 2011), are amongst the most complete *Fox* gene repertoires for a spiralian reported to date, and thus helps to clarify the ancestral *Fox* gene complement in Spiralia and Lophotrochozoa (Marlétaz *et al*. 2019), as well as the evolution of certain *Fox* gene classes in this animal group. Indeed, our study demonstrates that *O. fusiformis* and the brachiopod *Lingula unguis* have *bona fide foxT* genes, revealing that this recently described *Fox* gene class is more ancient that initially thought and not limited or specific to Panarthropoda (Schomburg *et al*. 2022). Therefore, all 21 *Fox* gene classes present in *O. fusiformis*, together with the missing *foxE* and *foxI* classes, were present in the last common spiralian and annelid ancestor, thus setting a reference complement for subsequent studies on the diversification of *Fox* genes in these animal clades.

Our study supports that lineage-specific losses and expansions are common in the evolution of the *Fox* genes in Spiralia. While a small number of *Fox* gene classes have been independently lost in some annelid lineages (e.g. *foxE* and *foxI* in *O. fusiformis*, *foxI* and *foxT* in *C. teleta*), our study supports that a full repertoire of *Fox* genes was present in the last common annelid ancestor (fig. 2). This contrasts with Mollusca (or at least Conchifera), which has experienced ancestral losses of two classes, namely *foxI* and *foxQ1*, as well as independent lineage-restricted losses (e.g. *foxG* and *foxM* in *C. gigas*, *foxO* in *Lottia gigantea*) (Shimeld *et al.* 2010a; Yang *et al.* 2014). Consistent with its faster rate of molecular evolution, the platyhelminth *S. mediterranea* has a more degraded *Fox* gene repertoire, with up to nine *Fox* classes missing (*foxAB*, *foxB*, *foxE*, *foxH*, *foxL2/3*, *foxQ1*, *foxJ2/3*, *foxM* and *foxN1/4*) (Pascual-Carreras *et al*. 2021). Notably, and as discussed below, expansions of the *foxQ2* class are common in Annelida and Mollusca, with some lineages, including *O. fusiformis*, exhibiting large lineage-specific expansions. Future work on the expression and function of *Fox* genes across spiralian lineages will help to clarify how these expansions and losses impact developmental programs and the diversification of body plans in Spiralia.

Gene architecture differences between clade I and II and the ancestral chromosomal linkage of the *foxC*, *foxF*, *foxL1* and *foxQ1* classes characterize the evolution of *Fox* genes (Larroux *et al*. 2008; Shimeld *et al.* 2010a; Schomburg *et al*. 2022). While our gene architecture data generally support the previous observations that the clade I of *Fox* genes are intronless, *O. fusiformis*—as also observed in the annelid *C. teleta*—does not exhibit a *Fox* gene cluster involving *foxC*, *foxF*, *foxL1* and *foxQ1* (Shimeld *et al.* 2010a). This contrasts with the overall conservation of gene macrosynteny in *O. fusiformis* (Martín-Zamora *et al.* 2022) and suggests that some intra-chromosomal rearrangements might have happened in this species. Despite the lack of a cluster organization, however, these genes retain a mesodermal expression in *O. fusiformis* (at least for *foxC*, *foxF*, *foxL1*; see below) (Martín-Durán *et al*. 2016), as observed in *C. teleta* and other spiralians (Shimeld *et al.* 2010a). Therefore, our study challenges the hypotheses that the concerted mesodermal co-expression of *foxC*, *foxF*, *foxL1* and *foxQ1* genes was the selective pressure for maintaining their cluster integrity (Shimeld *et al.* 2010a; Shimeld *et al.* 2010b), suggesting instead that more local, gene-specific regulation is responsible for their expression dynamics.

### The Expression Dynamics of the *Fox* Gene Complement in *O. fusiformis*

By combining new and previously reported temporal and spatial expression data in *O. fusiformis*, our study provides a comprehensive view of the developmental dynamics of *Fox* genes in this annelid species, suggesting conserved and potentially new developmental roles for certain *Fox* classes. In most animals studied to date, including annelids, molluscs, brachiopods, phoronid, bryozoans, planarians, and nemertean species, *foxA* is a key effector of foregut formation and a marker of endodermal tissues (Lartillot *et al*. 2002; Arenas-Mena 2006; Boyle and Seaver 2008, 2010; Jr Kai Yu *et al*. 2008; Fuchs *et al*. 2011; Adler *et al*. 2014; Fritzenwanker *et al*. 2014; Martín-Durán *et al*. 2015, 2016; Perry *et al*. 2015; Vellutini *et al*. 2017; Kwak *et al*. 2018; Andrikou *et al*. 2019; Kostyuchenko *et al*. 2019; Pascual-Carreras *et al*. 2021). In *O. fusiformis*, *foxA* is first detected in the vegetal macromeres at the blastula stage and later observed in the gastrula endoderm (peak of expression) and in the mouth and midgut of the developing larvae (Martín-Durán *et al*. 2016), thus supporting the role of the *foxA* class in endoderm and gut formation in animals. Similarly, the gene *foxJ1* is involved in ciliogenesis in a number of animals (Xianwen Yu et al. 2008; Choksi *et al*. 2014), including during the formation of the prototroch in the annelid *P. dumerilii* (Marlow *et al*. 2014). In *O. fusiformis*, *foxJ1* is expressed soon after gastrulation in the presumptive prototroch precursors and later on in the heavily ciliated cell types of the larva (the apical organ and prototroch) (Carrillo-Baltodano *et al*. 2021), reinforcing a conserved role of this *Fox* gene in the development of ciliated organs in Annelida and metazoans in general.

Existing gene expression data also support a conserved role of several *Fox* gene classes in mesoderm development in *O. fusiformis*. The *foxH* class regulates mesoderm development and embryonic organizing activity in vertebrates (Pogoda *et al*. 2000; Hoodless *et al*. 2001; Kofron *et al*. 2004), and it is downstream of the embryonic organizer and likely involved in mesoderm and posterodorsal development in *O. fusiformis* (Seudre *et al*. 2022), with a similar temporal expression dynamic reported in the oyster *C. gigas* (Yang *et al.* 2014). Similarly, the temporal and spatial expression domains of *foxL1*, *foxC* and *foxF* support their role in mesoderm formation in *O. fusiformis* (Martín-Durán *et al*. 2016), as described in other spiralians and metazoans (Wotton *et al*. 2008; Shimeld *et al.* 2010a; Wotton and Shimeld 2011; Fritzenwanker *et al*. 2014), albeit they do not retain their linked chromosomal position in this annelid species. Notably, *foxQ1* which is absent from all molluscan species studied to date, is expressed in the stomodeum and pharynx in *C. teleta* (Shimeld *et al.* 2010a; Yang *et al.* 2014; Wu *et al*. 2020). However, *foxQ1* is only expressed at the juvenile stage in *O. fusiformis*, suggesting that the role of this *Fox* gene class might differ between annelid and spiralian species.

The expression of *Fox* genes in other annelids and spiralians combined with the temporal dynamics of orthologs in *O. fusiformis* provide evidence of the potential roles of certain *Fox* gene classes in this annelid species. For instance, *foxD* is involved in myogenesis and ventral patterning in the annelid *P. dumerilii*, the platyhelminth *S. mediterranea* and the brachiopod *T. transversa* (Lauri *et al*. 2014; Passamaneck *et al*. 2015; Pascual-Carreras *et al*. 2021) and it becomes expressed after gastrulation and specially during organogenesis and initiation of myogenesis in *O. fusiformis* (Carrillo-Baltodano *et al*. 2021). The *foxO* class is a regulator of cell death in the planarian *S. mediterranea* (Pascual-Carreras *et al*. 2021) and an effector of cell division during early cleavage in the annelid *H. austinensis* (Kwak *et al*. 2018). In *O. fusiformis*, *foxO* is also expressed maternally and during early cleavage, as well as during embryonic periods with active cell turnover (Carrillo-Baltodano *et al*. 2021). The expression of the *foxAB* class has been only studied in the annelid *C. teleta*, which has a single ortholog that becomes expressed in a unique D-quadrant cell during early cleavage and is later involved in ectoderm differentiation and foregut formation (Boyle *et al*. 2014). *Owenia fusiformis* has instead two *foxAB* paralogs, one (*foxAB-1*) exclusively expressed at the time of the specification of the organizer cell at 5 hours post-fertilization and a second one (*foxAB-2*) that peaks at the gastrula stage before gradually fading away during larval development. Although further expression analyses are needed, we speculate that the two paralogs in *O. fusiformis* could play similar roles than those described in *C. teleta*, with *foxAB-1* acting during the axial body patterning and *foxAB-2* being involved in ectoderm and foregut formation later in embryogenesis.

Our comprehensive developmental time course of the *Fox* genes in *O. fusiformis* also uncovered dynamics of expression for *Fox* gene classes for which there is little understanding of their roles during annelid and spiralian embryogenesis. For instance, *foxT* is mostly expressed in the oocyte, early development and pre-gastrulation, which contrast with the generally late developmental expression reported in arthropods (Lin *et al*. 2021; Janssen *et al*. 2022). The genes *foxJ2/3*, *foxM*, and *foxN1/4* also show maternal expression, and for *foxM* and *foxN1/4* their expression lasts until the blastula stage (Supplementary fig. 5, Supplementary material online), where they are either expressed ubiquitously (*foxM*) or at the apical pole (*foxN1/4*), suggesting that they might be potential regulators of early cleavage and/or cell fate specification in this species. The genes *foxG, foxN2/3* and *foxP* are expressed at the time of the specification of the embryonic organizer in *O. fusiformis* (Seudre *et al*. 2022) (fig. 4*A*), and could therefore be involved in the establishment of the embryonic polarity and body plan in this annelid, as suggested by the expression of *foxN2/3* and *foxP* in the endoderm and apical/anterior ectoderm, respectively (Supplementary fig. 5, Supplementary material online). Finally, some *Fox* genes are restricted to either the larva (*foxK*) or the juvenile (*foxB, foxL2/3* and *foxQ1*), with expression data suggesting that at least *foxK* might be involved in the development of antero-ventral and oral ectoderm (Supplementary fig. 5, Supplementary material online). Altogether, our study sets the stage for further expression and functional studies of *Fox* genes in *O. fusiformis* and spiralian embryogenesis, which ultimately will help to better understand the plasticity and development roles of this major family of transcription factors in animal development and evolution.

### The Complex Evolutionary History of *foxQ2* Genes

The *foxQ2* class often comprises at least two paralogs in many cnidarian and bilaterian lineages studied to date, except in Tetrapoda, which lost this *Fox* gene class (Mazet *et al*. 2003; Yu *et al*. 2003; Chevalier *et al*. 2006; Santagata *et al*. 2012; Sinigaglia *et al*. 2013; Fritzenwanker *et al*. 2014; Marlow *et al*. 2014). Notably, *foxQ2* genes show a remarkable conservation of their expression patterns across phylogenetically distant animal lineages. In deuterostomes like the cephalochordate *Branchiostoma floridae*, the echinoderm *Strongylocentrotus purpuratus* and the hemichordate *Saccoglossus kowalevskii, foxQ2* genes are expressed in the apical pole during embryogenesis (Fritzenwanker *et al*. 2014; Range and Wei 2016), and *foxQ2* genes also play central roles in anterior development in the insects *D. melanogaster* and *Tribolium castaneum*, and the spider *Parasteatoda tepidariorum* (Lee and Frasch 2004; Kitzmann *et al*. 2017; Schacht *et al*. 2020). In the cnidarians *Nematostella vectensis* and *Clytia hemisphaerica*, *foxQ2* genes are expressed in and required for the proper development of the aboral pole, in support for the—still debated—homology between the cnidarian aboral pole and the bilaterian anterior pole (Chevalier *et al*. 2006; Sinigaglia *et al*. 2013). In Spiralia, *foxQ2* genes share an apical expression in the annelid *P. dumerilii* and the brachiopod *T. transversa* (Santagata *et al*. 2012; Marlow *et al*. 2014), which, as our study shows, is consistent with the majority of expression domains for *foxQ2* genes observed in the annelid *O. fusiformis*. Therefore, *foxQ2* genes appear to participate in ancient and broadly conserved genetic programs for apical and axial patterning in metazoans.

The consistent expression of *foxQ2* genes in apical territories contrasts, however, with the complex phylogenetic pattern of evolution of this class of *Fox* genes. As our study reveals, expansions of the *foxQ2* class are common in Spiralia, and specially in Annelida, with 11 paralogs in *O. fusiformis*, and 10 and 13 in the vestimentiferans *L. luymesi* and *P. echinospica*, respectively. While many of these paralogs probably emerged from species-specific expansions (e.g. *foxQ2-1*, *foxQ2-4*, *foxQ2-5*, *foxQ2-6*, and *foxQ2-9* in *O. fusiformis*; and the cluster of vestimentiferan *foxQ2* paralogs with an N-terminal EH-i-like motif) other paralogs might have a more ancient origin, tracing back to Annelida (e.g. *foxQ2-1* in *C. teleta* and *foxQ2-11* in *O. fusiformis*, both expressed maternally) and even Spiralia (e.g. *foxQ2-2* in *C. teleta* and *foxQ2-4* in *M. yessoensis*). Notably, however, nearly all bilaterian and cnidarian lineages retain at least a copy (two in the annelids *O. fusiformis* and *D. gyrociliatus*) of a subclass of *foxQ2* genes with a C-terminal EH-i-like motif. In *O. fusiformis,* these two genes (*foxQ2-2* and *foxQ2-3*) are controlled by the ERK1/2 signaling that establishes the axial polarity of the embryo and consequently show a narrow peak of expression at the animal pole at the time of the specification of the organizer cell at five hours post fertilization (Seudre *et al*. 2022). Based on these observations, we propose a model in which an ancestral *foxQ2* gene containing a C-terminal EH-i-like motif originated in the last common ancestor to Cnidaria and Bilateria, followed by independent expansions in certain bilaterian and cnidarian lineages and fast divergence of the new copies, which tended to lose the EH-i-like motif. Despite these duplications, however, the new paralogs did not generally acquire radically different functions (i.e. neofunctionalization) but rather retain a role in aboral/apical/anterior development, and evolved temporal, spatial and regulative specialization of their expression (i.e. subfunctionalization), as observed for example in *O. fusiformis* and the hemichordate *S. kowalevskii* (Fritzenwanker *et al*. 2014). Further analyses of the gene regulatory network associated with *foxQ2* genes in a broader range of metazoans, especially in spiralians with multiple copies, and role of the EH-i-like motif will contribute to uncover the evolutionary history and developmental consequences of *foxQ2* expansions during animal diversification.

Altogether, our study informs the evolution, temporal, and spatial expression of the largely conserved *Fox* gene repertoire in the oweniid annelid *O. fusiformis*. Our findings provide valuable information to reconstruct the ancestral complement of these core developmental regulators in Spiralia and to continue unravelling the embryological role and contribution of this major family of transcription factors to the evolution of animal body plans.

## Materials and Methods

### Animal Husbandry and Embryo Collection

Adult specimens of *Owenia fusiformis* Delle Chiaje, 1844 were collected and shipped to London from the coast near the Station Biologique de Roscoff (France) during their reproductive season (May to July). In the lab, animals were kept in aquaria with mud and artificial seawater (ASW) at 15°C. *In vitro* fertilizations were conducted as previously described (Martín-Durán *et al*. 2016; Carrillo-Baltodano *et al*. 2021) and embryos were kept in glass bowls at 19°C until they reach the desired developmental stage. Larval stages were relaxed in 8% MgCl2 and all embryonic samples fixed in 4% formaldehyde in sea water (or MgCl2, for larvae) for 1 h at room temperature. After washing the fixative with phosphate buffer saline (PBS) supplemented with 0.1% Tween-20, embryos and larvae were dehydrated to 100% methanol and stored at −20°C.

### Identification of *Forkhead* Genes and Orthology Assignment

Candidate *Fox* genes for *O. fusiformis* were initially retrieved from the functional annotation of its genome assembly (European Nucleotide Archive, accession number: GSE184126) (Martín-Zamora *et al.* 2022), and the *foxC* sequence was obtained from a previous study (Martín-Durán *et al*. 2016). The annotated *Fox* sequences from spiralians (*C. teleta*, *C. gigas*, *L. gigantea*, *M. yessoensis*, *Terebratalia transversa, Crepidula fornicata, L. unguis*), ecdysozoans (*D. melanogaster, Strigamia maritima, Caenorhabditis elegans, T. castaneum)*, deuterostomes (*Homo sapiens*, *Danio rerio, Mus musculus, Branchiostoma lanceolatum, S. kowalevskii, S. purpuratus*) and the cnidarian *N. vectensis* were identified by mining published transcriptomes and databases (Supplementary figs. 1 and 6, Supplementary material online). In addition, the genomes of the gene models for the annelids *O. fusiformis*, *D. gyrociliatus*, *C. teleta*, *E. andrei*, *H. robusta*, *S. benedicti*, *L. luymesi* and *P. echninospica*, as well as the gene models for *P. dumerilii* were mined for forkhead-containing proteins through Hidden Markov model (HMM) searches using the Pfam HMM profile for the forkhead domain (PF00250). Multiple protein alignments (Supplementary fig. 1, Supplementary material online; Supplementary file 1, Supplementary material online) were performed with MAFFT v.7 (Katoh and Standley 2013) with the L-INS-i strategy, trimming the forkhead domain as reported in the HMM profile. Maximum likelihood trees were constructed with IQTree v.2.2.0-beta (Minh *et al*. 2020) with automatic identification of the model of protein evolution and 1000 rapid bootstraps. Bayesian reconstructions in MrBayes v.3.2.7a (Ronquist and Huelsenbeck 2003) were also performed using the CAT model of protein evolution and two runs with four chains (one cold, three hot) run for 50,000,000 generations. Resulting trees (Supplementary figs. 2 and 3, Supplementary material online) were visualized and edited with FigTree (https://github.com/rambaut/figtree/).

### Manual Curation and Genomic Structure of *Fox* Genes

Short Illumina RNA-seq reads from a developmental time course aligned to the genome and a *de novo* assembled transcriptome (Martín-Zamora *et al.* 2022) were used to manually curate the automatic annotation and produce full length transcripts for *foxN1/4* (genes OFUSG25429.1 and OFUSG25430.1 were merged into a single gene), *foxK* (genes OFUSG10014.1 and OFUSG10013.1 were merged into a single gene), *foxQ2-10* (genes OFUSG24642.1 and OFUSG24641.1 were merged into a single gene) and the *Fox* orphan-2 gene (genes OFUSG25458.1 and OFUSG25457.1 were merged into a single gene). The exon and intron positions, as well as the chromosomal location for each *Fox* gene were determined based on the gene annotation of the genome assembly of *O. fusiformis* (Martín-Zamora *et al.* 2022) using the Integrative Genomics Viewer (Robinson *et al*. 2011). The position of the Forkhead domain within each *Fox* gene was determined using the Conserved Domain Database (CDD/SPARCLE) (Lu *et al*. 2020). The genomic architecture of the *Fox* genes was visualized using the online software IBS (Liu *et al*. 2015) and transferred to Illustrator Creative Cloud 2022 (Adobe).

### Phylogenetic Analysis of the *foxQ2* Family

To study the evolution of the FoxQ2 family in Spiralia, we retrieved sequences from spiralians (*C. teleta, C. gigas, D. gyrociliatus, E. andrei, H. robusta, L. gigantea, L. luymesi, M. yessoensis, P. dumerilii, P. echinospica, S. benedicti*), ecdysozoans (*D. melanogaster, N. vetripennis, P. humanus, T. castaneum*), deuterostomes (*Oryzias latipes, S. kowalevskii, S. purpuratus, B. floridae*) and cnidarians (*C. hemisphaerica, Hydra vulgaris, N. vectensis*) from published genomes, transcriptomes and databases. For all genes, membership to the *foxQ2* family was manually confirmed using the conserved domain database CDD/SPARCLE (Lu *et al*. 2020) and identifying the presence of a full FoxQ2 specific domain (Accession number CD20035) within the sequences. Some genes previously misannotated and assigned to other *Fox* gene classes were renamed as *foxQ2*. To identify the EH-I like domain in the *foxQ2* genes, we generated a EH-1-like motif position-specific scoring matrix using STREME v 5.4.1 (Bailey 2021), with a motif width of 7 to 10 amino acid and retaining motifs with *P*-value < 0.05, and confirmed the identified motif by comparison with results obtained by (Yaklichkin *et al*. 2007) (fig. 4*B*). The position and the presence of the EH-like motifs within the FoxQ2 sequences were determined by inputting the EH-i-like domain matrix into FIMO v 5.4.1 (Grant *et al*. 2011), matching motifs with a q-value < 0.05. For phylogenetic analyses, multiple protein alignments were performed with MAFFT v.7 as explained above and trees were reconstructed from a set of sequences selected with a Q.insect amino acid replacement matrix (Minh *et al*. 2021) to account for transition rates, the gamma distribution with four categories (G4) (Yang 1994) to describe sites evolution rates, and an optimization of amino acid frequencies using maximum likelihood in IQ-TREE v.2.1.2 (Minh *et al*. 2020). A thousand ultrafast bootstraps were used to extract branch support values and posterior probabilities were obtained through Bayesian reconstruction in MrBayes v.3.2.7a (Ronquist and Huelsenbeck 2003) as described above using the general time reversible model as a prior and 50,000,000 generations.

### Gene Expression Developmental Time Course

Stage-specific RNA-seq data covering 14 time-points, from the unfertilized oocyte to the juvenile stage (Martín-Zamora *et al.* 2022) were used to retrieve gene expression dynamics for all *Fox* genes in *O. fusiformis*. The transcriptomes of multiple developmental stages of the molluscs *C. gigas* and *M. yessoensis* where retrieved from Gene Expression Omnibus (accession number GSE31012) (Zhang *et al*. 2012) and the Short Read Archive database (accession numbers SRX1026991, SRX2238787 to SRX2238809, SRX2250256 to SRX2250259, SRX2251047, SRX2251049, SRX2251056, SRX2251057 and SRX2279546) (Wang *et al*. 2017), respectively (Supplementary table 3, Supplementary material online). The developmental expression profiles of *foxQ2* paralogs in *C. teleta* were computed using stage-specific RNA-seq data (Martín-Zamora *et al.* 2022) (Supplementary table 4, Supplementary material online). For all four species, if more than one sample were collected for a given developmental stage, the values of expression of the different replicates were averaged. The timing of sample collection and the number of replicates for each species and stages is detailed in Supplementary Table 3, Supplementary material online. Heatmaps were generated using the package pheatmap v.1.0.12 available in R, where color intensity shows the z-score value for each candidate genes (blue: low expression, red: high expression) (Kolde 2015).

### Gene Isolation and Whole Mount *in Situ* Hybridization

*foxQ2* genes, as well as *foxJ1*, *foxK*, *foxM*, *foxN1/4*, *foxN2/3* and *foxP* in *O. fusiformis* were amplified using gene specific primers, producing DNA templates for riboprobe synthesis by successive rounds of nested PCR on cDNA obtained from mixed developmental stages as initial template. Riboprobes were synthesized with the T7 enzyme following manufacturer's recommendations (Ambion's MEGAscript kit, #AM1334) and stored in hybridization buffer at a concentration of 50 ng μ/l at −20°C. Single colorimetric *in situ* hybridization of embryos and mitraria larvae were performed following an established protocol (Martín-Durán *et al*. 2016; Carrillo-Baltodano *et al*. 2021; Seudre *et al*. 2022).

### Imaging

Representative embryos from colorimetric whole mount *in situ* hybridization were cleared in 70% glycerol in PBS and imaged with a Leica DMRA2 upright epifluorescent microscope equipped with an Infinity5 camera (Lumenera), using bright field, differential interference contrast optics. Brightness/contrast and color balance were adjusted using Pixelmator Pro (v. 2.0.3) and applied to the whole image, not parts. Final figure panels were designed using Illustrator Creative Cloud 2022 (Adobe).

## Supplementary Material

evac139_Supplementary_Data

## Data Availability

The data underlaying this article are available in the article and in its online Supplementary material.

## References

[evac139-B1] Adler CE , Seidel CW, McKinney SA, Sánchez Alvarado A. 2014. Selective amputation of the pharynx identifies a FoxA-dependent regeneration program in planaria. eLife. 3:1–22. 10.7554/eLife.02238 PMC3985184 24737865

[evac139-B2] Andrikou C , Passamaneck YJ, Lowe CJ, Martindale MQ, Hejnol A. 2019. Molecular patterning during the development of *Phoronopsis harmeri* reveals similarities to rhynchonelliform brachiopods. Evodevo. 10:1–15. 31867094 10.1186/s13227-019-0146-1 PMC6907167

[evac139-B3] Arenas-Mena C . 2006. Embryonic expression of *HeFoxA1* and *HeFoxA2* in an indirectly developing polychaete. Dev Genes Evol. 216:727–736. 17031669 10.1007/s00427-006-0099-y

[evac139-B4] Bailey TL . 2021. STREME: accurate and versatile sequence motif discovery. Bioinformatics 37:2834–2840. 10.1093/bioinformatics/btab203 PMC8479671 33760053

[evac139-B5] Benayoun BA , Caburet S, Veitia RA. 2011. Forkhead transcription factors: key players in health and disease. Trends Genet. 27:224–232. 21507500 10.1016/j.tig.2011.03.003

[evac139-B6] Boyle MJ , Seaver EC. 2008. Developmental expression of *foxA* and *gata* genes during gut formation in the polychaete annelid, *Capitella* sp. I. Evol Dev. 10:89–105. 18184360 10.1111/j.1525-142X.2007.00216.x

[evac139-B7] Boyle MJ , Seaver EC. 2010. Expression of *FoxA* and *GATA* transcription factors correlates with regionalized gut development in two lophotrochozoan marine worms: *Chaetopterus* (Annelida) and *Themiste lageniformis* (Sipuncula). EvoDevo. 1:2. 10.1186/2041-9139-1-2 PMC2938726 20849645

[evac139-B8] Boyle MJ , Yamaguchi E, Seaver EC. 2014. Molecular conservation of metazoan gut formation: evidence from expression of endomesoderm genes in *Capitella teleta* (Annelida). EvoDevo. 5:39. 10.1186/2041-9139-5-39 PMC4407770 25908956

[evac139-B9] Carlsson P , Mahlapuu M. 2002. Forkhead transcription factors: key players in development and metabolism. Dev Biol. 250:1–23. 12297093 10.1006/dbio.2002.0780

[evac139-B10] Carrillo-Baltodano AM , et al 2021. Early embryogenesis and organogenesis in the annelid *Owenia fusiformis*. EvoDevo. 12:5. 10.1186/s13227-021-00176-z PMC8111721 33971947

[evac139-B11] Chevalier S , Martin A, Leclère L, Amiel A, Houliston E. 2006. Polarised expression of *FoxB* and *FoxQ2* genes during development of the hydrozoan *Clytia hemisphaerica*. Dev Genes Evol. 216:709–720. 17021866 10.1007/s00427-006-0103-6

[evac139-B12] Choksi SP , Lauter G, Swoboda P, Roy S. 2014. Switching on cilia: transcriptional networks regulating ciliogenesis. Development. 141:1427–1441. 10.1242/dev.074666 24644260

[evac139-B13] Chou HC , Acevedo-Luna N, Kuhlman JA, Schneider SQ. 2018. PdumBase: a transcriptome database and research tool for *Platynereis dumerilii* and early development of other metazoans. BMC Genomics. 19:618. 30115014 10.1186/s12864-018-4987-0 PMC6097317

[evac139-B14] Clark KL , Halay ED, Lai E, Burley SK. 1993. Co-crystal structure of the HNF-3/fork head DNA-recognition motif resembles histone H5. Nature. 364:412–420. 8332212 10.1038/364412a0

[evac139-B15] Coronel-córdoba P , Molina MD, Cardona G, Fraguas S. 2022. *Foxk1* is required for ectodermal cell differentiation during planarian regeneration. Front Cell Dev Biol. 10:808045. 35273960 10.3389/fcell.2022.808045 PMC8901602

[evac139-B16] Fritzenwanker JH , Gerhart J, Freeman RM, Lowe CJ. 2014. The Fox/Forkhead transcription factor family of the hemichordate *Saccoglossus kowalevskii*. EvoDevo. 5:17. 10.1186/2041-9139-5-17 PMC4077281 24987514

[evac139-B17] Fuchs J , Martindale MQ, Hejnol A. 2011. Gene expression in bryozoan larvae suggest a fundamental importance of pre-patterned blastemic cells in the bryozoan life-cycle. EvoDevo. 2:13. 10.1186/2041-9139-2-13 PMC3133996 21645327

[evac139-B18] Gąsiorowski L , Hejnol A. 2020. *Hox* gene expression during development of the phoronid *Phoronopsis harmeri*. EvoDevo. 11:2. 32064072 10.1186/s13227-020-0148-z PMC7011278

[evac139-B19] Golson ML , Kaestner KH. 2016. Fox transcription factors: from development to disease. Development. 143:4558–4570. 27965437 10.1242/dev.112672 PMC5201025

[evac139-B20] Grant CE , Bailey TL, Noble WS. 2011. FIMO: scanning for occurrences of a given motif. Bioinformatics. 27:1017–1018. 21330290 10.1093/bioinformatics/btr064 PMC3065696

[evac139-B21] Hannenhalli S , Kaestner KH. 2009. The evolution of fox genes and their role in development and disease. Nat Rev Genet. 10:233–240. 19274050 10.1038/nrg2523 PMC2733165

[evac139-B22] He B , et al 2019. An ancestral apical brain region contributes to the central complex under the control of *foxQ2* in the beetle *Tribolium*. eLife. 8:1–29. 10.7554/eLife.49065 PMC6837843 31625505

[evac139-B23] Hoodless PA , et al 2001. Foxh1 (Fast) functions to specify the anterior primitive streak in the mouse. Genes Dev. 1:1257–1271. 10.1101/gad.881501 PMC313796 11358869

[evac139-B24] Hunnekuhl VS , Akam M. 2014. An anterior medial cell population with an apical-organ-like transcriptional profile that pioneers the central nervous system in the centipede *Strigamia maritima*. Dev Biol. 396:136–149. 25263198 10.1016/j.ydbio.2014.09.020

[evac139-B25] Jackson BC , Carpenter C, Nebert DW, Vasiliou V. 2010. Update of human and mouse forkhead box (FOX) gene families. Hum Genomics. 4:345–352. 20650821 10.1186/1479-7364-4-5-345 PMC3500164

[evac139-B26] Janssen R . 2017. Comparative analysis of gene expression patterns in the arthropod labrum and the onychophoran frontal appendages, and its implications for the arthropod head problem. EvoDevo. 8:1. 28053697 10.1186/s13227-016-0064-4 PMC5209905

[evac139-B27] Janssen R , Schomburg C, Prpic NM, Budd GE. 2022. A comprehensive study of arthropod and onychophoran Fox gene expression patterns. PLoS ONE. 17(7):e0270790. 35802758 10.1371/journal.pone.0270790 PMC9269926

[evac139-B28] Kaestner KH , Knöchel W, Martínez DE. 2000. Unified nomenclature for the winged helix/forkhead transcription factors. Genes Dev. 14:142–146. 10702024

[evac139-B29] Katoh K , Standley DM. 2013. MAFFT multiple sequence alignment software version 7: improvements in performance and usability. Mol Biol Evol. 30:772–780. 23329690 10.1093/molbev/mst010 PMC3603318

[evac139-B30] Kaufmann E , Knöchel W. 1996. Five years on the wings of fork head. Mech Dev. 57:3–20. 8817449 10.1016/0925-4773(96)00539-4

[evac139-B31] Kitzmann P , Weißkopf M, Schacht MI, Bucher G. 2017. A key role for foxQ2 in anterior head and central brain patterning in insects. Dev. 144:2969–2981. 10.1242/dev.147637 PMC5592812 28811313

[evac139-B32] Kofron M , et al 2004. New roles for FoxH1 in patterning the early embryo. Development. 131:5065–5078. 15459100 10.1242/dev.01396

[evac139-B33] Kostyuchenko RP , Kozin VV, Filippova NA, Sorokina EV. 2019. *Foxa* expression pattern in two polychaete species, *Alitta virens* and *Platynereis dumerilii*: examination of the conserved key regulator of the gut development from cleavage through larval life, postlarval growth, and regeneration. Dev Dyn. 248:1–16. 10.1002/dvdy.7 30566266

[evac139-B34] Kwak HJ , Ryu KB, Medina Jiménez BI, Park SC, Cho SJ. 2018. Temporal and spatial expression of the Fox gene family in the leech *Helobdella austinensis*. J Exp Zool Part B Mol Dev Evol. 330:341–350. 10.1002/jez.b.22828 30280505

[evac139-B35] Larroux C , et al 2006. Developmental expression of transcription factor genes in a demosponge: insights into the origin of metazoan multicellularity. Evol Dev. 8:150–173. 16509894 10.1111/j.1525-142X.2006.00086.x

[evac139-B36] Larroux C , et al 2008. Genesis and expansion of metazoan transcription factor gene classes. Mol Biol Evol. 25:980–996. 18296413 10.1093/molbev/msn047

[evac139-B37] Lartillot N , Lespinet O, Vervoort M, Adoutte A. 2002. Expression pattern of *Brachyury* in the mollusc *Patella vulgata* suggests a conserved role in the establishment of the AP axis in Bilateria. Development. 129:1411–1421. 11880350 10.1242/dev.129.6.1411

[evac139-B38] Lauri A , et al 2014. Development of the annelid axochord: insights into notochord evolution. Science. 345:1356–1368. 10.1126/science.1253396 25214631

[evac139-B39] Lee HH , Frasch M. 2004. Survey of forkhead domain encoding genes in the drosophila genome: classification and embryonic expression patterns. Dev Dyn. 229:357–366. 14745961 10.1002/dvdy.10443

[evac139-B40] Li Y , et al 2016. Transcriptome sequencing and comparative analysis of ovary and testis identifies potential key sex-related genes and pathways in scallop *Patinopecten yessoensis*. Mar Biotechnol. 18:453–465. 10.1007/s10126-016-9706-8 27234819

[evac139-B41] Li Y , et al 2019. Genomic adaptations to chemosymbiosis in the deep-sea seep-dwelling tubeworm *Lamellibrachia luymesi*. BMC Biol. 17:1–14. 31739792 10.1186/s12915-019-0713-x PMC6862839

[evac139-B42] Li C , Tucker PW. 1993. DNA-binding properties and secondary structural model of the hepatocyte nuclear factor 3/fork head domain. Proc Natl Acad Sci U S A. 90:11583–11587. 8265594 10.1073/pnas.90.24.11583 PMC48028

[evac139-B43] Lin HY , et al 2021. The genetic network of Forkhead gene family in development of brown planthoppers. Biology (Basel). 10:867. 34571744 10.3390/biology10090867 PMC8469257

[evac139-B44] Liu W , et al 2015. IBS: an illustrator for the presentation and visualization of biological sequences. Bioinformatics. 31:3359–3361. 26069263 10.1093/bioinformatics/btv362 PMC4595897

[evac139-B45] Liu XL , Zhang ZF, Shao MY, Liu JG, Muhammad F. 2012. Sexually dimorphic expression of foxl2 during gametogenesis in scallop *Chlamys farreri*, conserved with vertebrates. Dev Genes Evol. 222:279–286. 22752442 10.1007/s00427-012-0410-z

[evac139-B46] Lu S , et al 2020. CDD/SPARCLE: the conserved domain database in 2020. Nucleic Acids Res. 48:D265–D268. 31777944 10.1093/nar/gkz991 PMC6943070

[evac139-B47] Magie CR , Pang K, Martindale MQ. 2005. Genomic inventory and expression of Sox and Fox genes in the cnidarian *Nematostella vectensis*. Dev Genes Evol. 215:618–630. 16193320 10.1007/s00427-005-0022-y

[evac139-B48] Marlétaz F , Peijnenburg KTCA, Goto T, Satoh N, Rokhsar DS. 2019. A new spiralian phylogeny places the enigmatic arrow worms among gnathiferans. Curr Biol. 29:312–318.e3. 30639106 10.1016/j.cub.2018.11.042

[evac139-B49] Marlow H , et al 2014. Larval body patterning and apical organs are conserved in animal evolution. BMC Biol. 12:1–17. 24476105 10.1186/1741-7007-12-7 PMC3939940

[evac139-B50] Martín-Durán JM , et al 2021. Conservative route to genome compaction in a miniature annelid. Nat Ecol Evol. 5:231–242. 33199869 10.1038/s41559-020-01327-6 PMC7854359

[evac139-B51] Martín-Durán JM , Passamaneck YJ, Martindale MQ, Hejnol A. 2016. The developmental basis for the recurrent evolution of deuterostomy and protostomy. Nat Ecol Evol. 1:0005. 10.1038/s41559-016-0005 28812551

[evac139-B52] Martín-Durán JM , Vellutini BC, Hejnol A. 2015. Evolution and development of the adelphophagic, intracapsular Schmidt's larva of the nemertean *Lineus ruber*. EvoDevo. 6:28. 26417429 10.1186/s13227-015-0023-5 PMC4584431

[evac139-B53] Martín-Zamora FM , et al 2022. Annelid functional genomics reveal the origins of bilaterian life cycles. bioRxiv. 2022.02.05.479245. 10.1038/s41586-022-05636-7 PMC9977687 36697830

[evac139-B54] Mazet F , et al 2006. An ancient Fox gene cluster in bilaterian animals. Curr Biol. 16:314–316. 10.1016/j.cub.2006.03.088 16682334

[evac139-B55] Mazet F , Yu JK, Liberles DA, Holland LZ, Shimeld SM. 2003. Phylogenetic relationships of the Fox (Forkhead) gene family in the Bilateria. Gene. 316:79–89. 14563554 10.1016/s0378-1119(03)00741-8

[evac139-B56] Minh BQ , et al 2020. IQ-TREE 2: new models and efficient methods for phylogenetic inference in the genomic era. Mol Biol Evol. 37:1530–1534. 32011700 10.1093/molbev/msaa015 PMC7182206

[evac139-B57] Minh BQ , Dang CC, Vinh LS, Lanfear R. 2021. QMaker: fast and accurate method to estimate empirical models of protein evolution. Syst Biol. 70:1046–1060. 33616668 10.1093/sysbio/syab010 PMC8357343

[evac139-B58] Paps J , et al 2012. A genome-wide view of transcription factor gene diversity in chordate evolution: less gene loss in amphioxus? Brief Funct Genomics. 11:177–186. 22441554 10.1093/bfgp/els012

[evac139-B59] Pascual-Carreras E , et al 2021. Analysis of Fox genes in *Schmidtea mediterranea* reveals new families and a conserved role of *Smed-foxO* in controlling cell death. Sci Rep. 11:1–18. 33536473 10.1038/s41598-020-80627-0 PMC7859237

[evac139-B60] Passamaneck YJ , Hejnol A, Martindale MQ. 2015. Mesodermal gene expression during the embryonic and larval development of the articulate brachiopod *Terebratalia transversa*. EvoDevo. 6:10 25897375 10.1186/s13227-015-0004-8 PMC4404124

[evac139-B61] Perry KJ , et al 2015. Deployment of regulatory genes during gastrulation and germ layer specification in a model spiralian mollusc *Crepidula*. Dev Dyn. 244:1215–1248. 26197970 10.1002/dvdy.24308

[evac139-B62] Pogoda HM , Solnica-krezel L, Driever W, Meyer D. 2000. The zebrafish forkhead transcription factor FoxH1/Fast1 is a modulator of nodal signaling required for organizer formation. Curr Biol. 10:1041–1049. 10996071 10.1016/s0960-9822(00)00669-2

[evac139-B63] Range RC , Wei Z. 2016. An anterior signaling center patterns and sizes the anterior neuroectoderm of the sea urchin embryo. Development. 143:1523–1533. 26952978 10.1242/dev.128165 PMC4909856

[evac139-B64] Robinson JT , et al 2011. Integrative genomics viewer. Nat Biotechnol. 29:24–26. 21221095 10.1038/nbt.1754 PMC3346182

[evac139-B65] Ronquist F , Huelsenbeck JP. 2003. Mrbayes 3: Bayesian phylogenetic inference under mixed models. Bioinformatics. 19:1572–1574. 12912839 10.1093/bioinformatics/btg180

[evac139-B66] Rouse GW , Pleijel F, Tilic E. 2022. Annelida. Oxford, UK: Oxford University Press.

[evac139-B67] Santagata S , Resh C, Hejnol A, Martindale MQ, Passamaneck YJ. 2012. Development of the larval anterior neurogenic domains of *Terebratalia transversa* (Brachiopoda) provides insights into the diversification of larval apical organs and the spiralian nervous system. EvoDevo. 3:3 22273002 10.1186/2041-9139-3-3 PMC3314550

[evac139-B68] Schacht MI , Schomburg C, Bucher G. 2020. *Six3* acts upstream of *foxQ2* in labrum and neural development in the spider *Parasteatoda tepidariorum*. Dev Genes Evol. 230:95–104. 32040712 10.1007/s00427-020-00654-9 PMC7128001

[evac139-B69] Schomburg C , Janssen R, Michael N. 2022. Phylogenetic analysis of forkhead transcription factors in the Panarthropoda. Dev Genes Evol. 232:39–48 35230523 10.1007/s00427-022-00686-3 PMC8918179

[evac139-B70] Seudre O , et al 2022. ERK1/2 is an ancestral organising signal in spiral cleavage. Nat Commun. 13:2286. 35484126 10.1038/s41467-022-30004-4 PMC9050690

[evac139-B71] Shao Y , et al 2020. Genome and single-cell RNA-sequencing of the earthworm *Eisenia andrei* identifies cellular mechanisms underlying regeneration. Nat Commun. 11:1–15. 32461609 10.1038/s41467-020-16454-8 PMC7253469

[evac139-B72] Shimeld SM , Boyle MJ, Brunet T, Luke GN, Seaver EC. 2010a. Clustered *Fox* genes in lophotrochozoans and the evolution of the bilaterian *Fox* gene cluster. Dev Biol. 340:234–248. 20096280 10.1016/j.ydbio.2010.01.015

[evac139-B73] Shimeld SM , Degnan B, Luke GN. 2010b. Evolutionary genomics of the *Fox* genes: origin of gene families and the ancestry of gene clusters. Genomics. 95:256–260. 19679177 10.1016/j.ygeno.2009.08.002

[evac139-B74] Simakov O , et al 2013. Insights into bilaterian evolution from three spiralian genomes. Nature. 493:526–531. 23254933 10.1038/nature11696 PMC4085046

[evac139-B75] Sinigaglia C , Busengdal H, Leclère L, Technau U, Rentzsch F. 2013. The bilaterian head patterning gene *six3/6* controls aboral domain development in a cnidarian. PLoS Biol. 11: e1001488. 10.1371/journal.pbio.1001488 PMC3586664 23483856

[evac139-B76] Sun Y , et al 2021. Genomic signatures supporting the symbiosis and formation of chitinous tube in the deep-sea tubeworm *Paraescarpia echinospica*. Mol Biol Evol. 38:4116–4134. 34255082 10.1093/molbev/msab203 PMC8476170

[evac139-B77] Teaniniuraitemoana V , et al 2015. Molecular signatures discriminating the male and the female sexual pathways in the pearl oyster *Pinctada margaritifera*. PLoS ONE. 10:1–20. 10.1371/journal.pone.0122819 PMC4376701 25815473

[evac139-B78] Tu Q , Brown CT, Davidson EH, Oliveri P. 2006. Sea urchin Forkhead gene family: Phylogeny and embryonic expression. Dev Biol. 300:49–62. 17081512 10.1016/j.ydbio.2006.09.031

[evac139-B79] Vellutini BC , Martín-durán JM, Hejnol A. 2017. Cleavage modification did not alter blastomere fates during bryozoan evolution. BMC Biol. 15:1–28. 28454545 10.1186/s12915-017-0371-9 PMC5408385

[evac139-B80] Wang S , et al 2017. Scallop genome provides insights into evolution of bilaterian karyotype and development. Nat Ecol Evol. 1:0120. 10.1038/s41559-017-0120 PMC10970998 28812685

[evac139-B81] Weigel D , et al 1989. The homeotic gene fork head encodes a nuclear protein and is expressed in the terminal regions of the drosophila embryo. Cell. 57:645–658. 2566386 10.1016/0092-8674(89)90133-5

[evac139-B82] Wotton KR , Mazet F, Shimeld SM. 2008. Expression of *FoxC*, *FoxF*, *FoxL1*, and *FoxQ1* genes in the dogfish *Scyliorhinus canicula* defines ancient and derived roles for fox genes in vertebrate development. Dev Dyn. 237:1590–1603. 18498098 10.1002/dvdy.21553

[evac139-B83] Wotton KR , Shimeld SM. 2006. Comparative genomics of vertebrate Fox cluster loci. BMC Genomics. 7:1–9. 17062144 10.1186/1471-2164-7-271 PMC1634998

[evac139-B84] Wotton KR , Shimeld SM. 2011. Analysis of lamprey clustered Fox genes: insight into Fox gene evolution and expression in vertebrates. Gene. 489:30–40. 21907770 10.1016/j.gene.2011.08.007

[evac139-B85] Wu S , et al 2020. Identification and expression profiles of Fox transcription factors in the Yesso scallop (*Patinopecten yessoensis*). Gene. 733:144387 10.1016/j.gene.2020.144387 31972308

[evac139-B86] Yaguchi S , Yaguchi J, Angerer RC, Angerer LM. 2008. A Wnt-FoxQ2-nodal pathway links primary and secondary axis specification in sea urchin embryos. Dev Cell. 14:97–107. 18194656 10.1016/j.devcel.2007.10.012

[evac139-B87] Yaklichkin S , Vekker A, Stayrook S, Lewis M, Kessler DS. 2007. Prevalence of the EH1 Groucho interaction motif in the metazoan Fox family of transcriptional regulators. BMC Genomics. 8:201. 17598915 10.1186/1471-2164-8-201 PMC1939712

[evac139-B88] Yang Z . 1994. Maximum likelihood phylogenetic estimation from DNA sequences with variable rates over sites: approximate methods. J Mol Evol. 39:306–314. 7932792 10.1007/BF00160154

[evac139-B89] Yang M , et al 2014. Phylogeny of forkhead genes in three spiralians and their expression in Pacific oyster *Crassostrea gigas*. Chin J Oceanol Limnol. 32(6):1207–1223.

[evac139-B90] Yu K Jr , et al 2008. The Fox genes of *Branchiostoma floridae*. Dev Genes Evol. 218:629–638. 18773219 10.1007/s00427-008-0229-9

[evac139-B91] Yu JK , Holland ND, Holland LZ. 2003. *AmphifoxQ2*, a novel winged helix/forkhead gene, exclusively marks the anterior end of the amphioxus embryo. Dev Genes Evol. 213:102–105. 12632180 10.1007/s00427-003-0302-3

[evac139-B92] Yu X , Ng CP, Habacher H, Roy S. 2008. Foxj1 transcription factors are master regulators of the motile ciliogenic program. Nat Genet. 40:1445–1453. 19011630 10.1038/ng.263

[evac139-B93] Zakas C , Harry ND, Scholl EH, Rockman MV. 2022. The genome of the poecilogonous annelid *Streblospio benedicti*. Genome Biol Evol. 14:evac008. 10.1093/gbe/evac008 PMC8872972 35078222

[evac139-B94] Zhang G , et al 2012. The oyster genome reveals stress adaptation and complexity of shell formation. Nature. 490:49–54. 22992520 10.1038/nature11413

