# Supplementary material for: The Fox Gene Repertoire in the Annelid Owenia fusiformis Reveals Multiple Expansions of the foxQ2 Class in Spiralia
Source: Genome Biol Evol. 2022 Sep 13;14(10):evac139. doi: 10.1093/gbe/evac139 (PMC9539403; doi:10.1093/gbe/evac139)
Supplement: evac139_Supplementary_Data [file evac139_supplementary_data.zip › FigureS6.pdf]

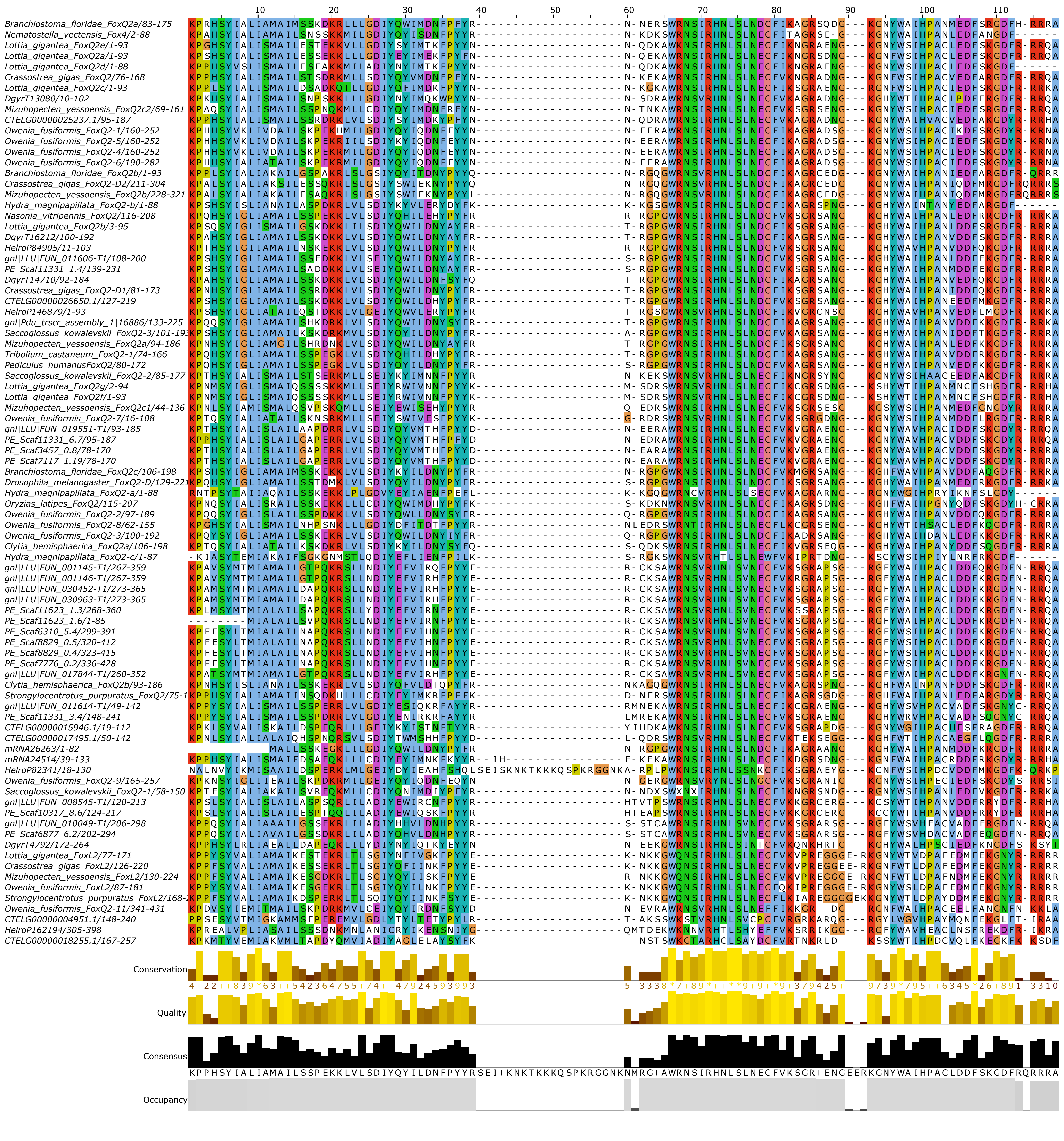

10
20
30
40
50
60
70
80
90
100
110
Branchiostoma_floridae_FoxQ2a/83-175
Nematostella_vectensis_Fox4/2-88
Lottia_gigantea_FoxQ2e/1-93
Lottia_gigantea_FoxQ2a/1-93
Lottia_gigantea_FoxQ2d/1-88
Crassostrea_gigas_FoxQ2/76-168
Lottia_gigantea_FoxQ2c/1-93
DgyrT13080/10-102
Mizuhopecten_yessoensis_FoxQ2c2/69-161
CTELG00000025237.1/95-187
Owenia_fusiformis_FoxQ2-1/160-252
Owenia_fusiformis_FoxQ2-5/160-252
Owenia_fusiformis_FoxQ2-4/160-252
Owenia_fusiformis_FoxQ2-6/190-282
Branchiostoma_floridae_FoxQ2b/1-93
Crassostrea_gigas_FoxQ2-D2/211-304
Mizuhopecten_yessoensis_FoxQ2b/228-321
Hydra_magnipapillata_FoxQ2-b/1-88
Nasonia_vitripennis_FoxQ2/116-208
Lottia_gigantea_FoxQ2b/3-95
DgyrT16212/100-192
HelroP84905/11-103
gnl|LLU|FUN_011606-T1/108-200
PE_Scaf11331_1.4/139-231
DgyrT14710/92-184
Crassostrea_gigas_FoxQ2-D1/81-173
CTELG00000026650.1/127-219
HelroP146879/1-93
gnl|Pdu_trscr_assembly_1|16886/133-225
Saccoglossus_kowalevskii_FoxQ2-3/101-193
Mizuhopecten_yessoensis_FoxQ2a/94-186
Tribolium_castaneum_FoxQ2-1/74-166
Pediculus_humanusFoxQ2/80-172
Saccoglossus_kowalevskii_FoxQ2-2/85-177
Lottia_gigantea_FoxQ2g/2-94
Lottia_gigantea_FoxQ2f/1-93
Mizuhopecten_yessoensis_FoxQ2c1/44-136
Owenia_fusiformis_FoxQ2-7/16-108
gnl|LLU|FUN_019551-T1/93-185
PE_Scaf11331_6.7/95-187
PE_Scaf3457_0.8/78-170
PE_Scaf7117_1.19/78-170
Branchiostoma_floridae_FoxQ2c/106-198
Drosophila_melanogaster_FoxQ2-D/129-221
Hydra_magnipapillata_FoxQ2-a/1-88
Oryzias_latipes_FoxQ2/115-207
Owenia_fusiformis_FoxQ2-2/97-189
Owenia_fusiformis_FoxQ2-8/62-155
Owenia_fusiformis_FoxQ2-3/100-192
Clytia_hemisphaerica_FoxQ2a/106-198
Hydra_magnipapillata_FoxQ2-c/1-87
gnl|LLU|FUN_001145-T1/267-359
gnl|LLU|FUN_001146-T1/267-359
gnl|LLU|FUN_030452-T1/273-365
gnl|LLU|FUN_030963-T1/273-365
PE_Scaf11623_1.3/268-360
PE_Scaf11623_1.6/1-85
PE_Scaf6310_5.4/299-391
PE_Scaf8829_0.5/320-412
PE_Scaf8829_0.4/323-415
PE_Scaf7776_0.2/336-428
gnl|LLU|FUN_017844-T1/260-352
Clytia_hemisphaerica_FoxQ2b/93-186
Strongylocentrotus_purpuratus_FoxQ2/75-1
gnl|LLU|FUN_011614-T1/49-142
PE_Scaf11331_3.4/148-241
CTELG00000015946.1/19-112
CTELG00000017495.1/50-142
mRNA26263/1-82
mRNA24514/39-133
HelroP82341/18-130
Owenia_fusiformis_FoxQ2-9/165-257
Saccoglossus_kowalevskii_FoxQ2-1/58-150
gnl|LLU|FUN_008545-T1/120-213
PE_Scaf10317_8.6/124-217
gnl|LLU|FUN_010049-T1/206-298
PE_Scaf6877_6.2/202-294
DgyrT4792/172-264
Lottia_gigantea_FoxL2/77-171
Crassostrea_gigas_FoxL2/126-220
Mizuhopecten_yessoensis_FoxL2/130-224
Owenia_fusiformis_FoxL2/87-181
Strongylocentrotus_purpuratus_FoxL2/168-2
Owenia_fusiformis_FoxQ2-11/341-431
CTELG00000004951.1/148-240
HelroP162194/305-398
CTELG00000018255.1/167-257
Conservation
Quality
Consensus
KPPHSYIALIAMAILSSPEKKLVLSDIYQYILDNFPYYRSEI+KNKTKKKQSPKRGGNKNMRG+AWRNSIRHNLSLNECFVKSGR+ENGEERKGNYWAIHPACLDDFSKGDFRQRRRA
Occupancy
