# Supplementary material for: The Fox Gene Repertoire in the Annelid Owenia fusiformis Reveals Multiple Expansions of the foxQ2 Class in Spiralia
Source: Genome Biol Evol. 2022 Sep 13;14(10):evac139. doi: 10.1093/gbe/evac139 (PMC9539403; doi:10.1093/gbe/evac139)
Supplement: evac139_Supplementary_Data [file evac139_supplementary_data.zip › SupplementaryTables_r.pdf]

Supplementary Table 1. The expression of *Fox* genes in Spiralia

Clade	Genes	Phyla	Species	Gene expression domain	Inferred roles	Reference
I	*foxA*	Annelida	*Hydroides elegans*	*(two paralogs; expression is mostly overlapping)*<br>Blastula: vegetal pole<br>Gastrula: blastopore, anterior lip of the blastopore	Gut formation and mesoderm development	Arenas-Mena, César. "Embryonic expression of HeFoxA1 and HeFoxA2 in an indirectly developing polychaete." Development genes and evolution 216.11 (2006): 727-736.
			Capitella teleta	Blastula: vegetal blastomeres<br>Gastrula: blastopore (anterior and posterior).<br>Larva: foregut, hindgut	Involved in foregut formation.	Boyle, M. J. & Seaver, E. C. Developmental expression of foxA and gata genes during gut formation in the polychaete annelid, Capitella sp. I. Evol. Dev. 10, 89-105 (2008)
			Chaetopterus variopedatus	Blastula: presumptive endoderm<br>Gastrula: Vegetal cells<br>Larvae: foregut and hindgut domains	Gut formation	Boyle, Michael J., and Elaine C. Seaver. "Expression of FoxA and GATA transcription factors correlates with regionalized gut development in two lophotrochozoan marine worms: Chaetopterus (Annelida) and Themiste lageniformis (Sipuncula)." EvoDevo 1.1 (2010): 1-18.
			Themiste lageniformis	Blastula: presumptive endoderm<br>Gastrula: Vegetal cells<br>Larvae: foregut and hindgut domains	Gut formation	Boyle, Michael J., and Elaine C. Seaver. "Expression of FoxA and GATA transcription factors correlates with regionalized gut development in two lophotrochozoan marine worms: Chaetopterus (Annelida) and Themiste lageniformis (Sipuncula)." EvoDevo 1.1 (2010): 1-18.
			Platynereis dumerilii	Cleavage: Three large blastomeres in quadrants A, C and B<br>Gastrulation:<br>Early protochophore: Stomodeal plate<br>Middle trochophore: Two bilaterally symmetric patches at the ventral episphere and two cells posterior to the prototroch at the ventrolateral sides of the hyposphere<br>Metatrochophore: Foregut primordium	Involved in foregut formation<br>Primary role in gastrulation morphogenesis<br>Marker of endoderm formation	Kostyuchenko, Roman P., et al. "FoxA expression pattern in two polychaete species, Alitta virens and Platynereis dumerilii: examination of the conserved key regulator of the gut development from cleavage through larval life, postlarval growth, and regeneration." Developmental Dynamics 248.8 (2019): 728-743.
			Alitta virens	Early trochophore: Individual cells around the blastopore and stomodeal plate<br>Mid-trochophore: Two bilaterally symmetric patches at the ventral episphere and two cells posterior to the prototroch at the ventrolateral sides of the hyposphere<br>Metatrochophore: Foregut primordium.	Involved in foregut formation<br>Primary role in gastrulation morphogenesis<br>Marker of endoderm formation	Kostyuchenko, Roman P., et al. "FoxA expression pattern in two polychaete species, Alitta virens and Platynereis dumerilii: examination of the conserved key regulator of the gut development from cleavage through larval life, postlarval growth, and regeneration." Developmental Dynamics 248.8 (2019): 728-743.
			Helobdella austinensis	*(two paralogs)*<br>Cleavage: absents<br>Organogenesis: Expressed in the prostomium and later in the proboscis.	Involved in foregut formation.	Kwak, Hee-Jin, et al. "Temporal and spatial expression of the Fox gene family in the Leech Helobdella austinensis." Journal of Experimental Zoology Part B: Molecular and Developmental Evolution 330.6-7 (2018): 341-350.

		Owenia fusiformis	Blastula: vegetal macromeres<br>Gastrula: endoderm, anterior lip blastopore<br>Larvae: mouth, midgut	Endodermal marker<br>Foregut marker	Martín-Durán, José M., et al. "The developmental basis for the recurrent evolution of deuterostomy and protostomy." Nature ecology & evolution 1.1 (2016): 1-10.<br>APA
	Mollusca	*Patella vulgata*	Cleavage: faint expression in 3A, 3B and 3C<br>Blastula: endodermal derivatives, except for 4D.<br>Gastrula: horseshoe pattern, Ectomesoderm,<br>Larvae: whole anterior mesodermal field	Gut formation<br>Mesoderm formation<br>Role in the establishment of anterior inductive patterning	Lartillot N, Le Gouar M, Adoutte A (2002) Expression patterns of fork headand goosecoid homologues in the mollusc Patella vulgata supports the ancestry of the anterior mesendoderm across Bilateria. Dev Genes Evol 212:551–561
		Crepidula fornicata	Cleavage: All A-D macromeres in regions adjacent to the nuclei<br>Gastrula: Cells of the blastopore lip<br>Larvae: Developing mouth and esophagus	Endodermal marker<br>Foregut marker	Perry, K. J., Lyons, D. C., Truchado-Garcia, M., Fischer, A. H., Helfrich, L. W., Johansson, K. B., ... & Henry, J. Q. (2015). Deployment of regulatory genes during gastrulation and germ layer specification in a model spiralian mollusc Crepidula. Developmental Dynamics, 244(10), 1215-1248.<br>ISO 690
	Brachiopoda	*Novocrania anomala*	Blastula: prospective endoderm in the gastral plate<br>Gastrula: archenteron wall<br>Larvae: gut, mouth and ventral ectoderm	Endodermal marker<br>Foregut marker	Martín-Durán, José M., et al. "The developmental basis for the recurrent evolution of deuterostomy and protostomy." Nature ecology & evolution 1.1 (2016): 1-10.<br>APA
		Terebratalia transversa	Blastula: prospective endoderm in the gastral plate<br>Gastrula: archenteron wall<br>Larvae: gut, mouth and anteroventral ectoderm	Endodermal marker<br>Foregut marker	Martín-Durán, José M., et al. "The developmental basis for the recurrent evolution of deuterostomy and protostomy." Nature ecology & evolution 1.1 (2016): 1-10.<br>APA
	Platyhelminth	*Schmidtea mediterranea*	*(two paralogs)*<br>Adult: FoxA-1 expressed inn the pharynx and its progenitors and FoxA-2 expressed in a dotted pattern all along the animal body, which could be neurons and/or epidermal cells.	Not determined	Pascual-Carreras, E., Herrera-Úbeda, C., Rosselló, M., Coronel-Córdoba, P., Garcia-Fernàndez, J., Saló, E., & Adell, T. (2021). Analysis of Fox genes in Schmidtea mediterranea reveals new families and a conserved role of Smed-foxO in controlling cell death. Scientific reports, 11(1), 1-18.
	Nemertea	*Lineus ruber*	Blastula: absent<br>Gastrula: sbsent<br>Larvae: mouth and pharynx<br>Juvenile: mouth, inner head, ventral side and posterior end of the endoderm	Endodermal marker<br>Foregut marker	Martín-Durán, J. M., Vellutini, B. C., & Hejnol, A. (2015). Evolution and development of the adelphophagic, intracapsular Schmidt's larva of the nemertean Lineus ruber. *Evodevo*, *6*(1), 1-18.
foxAB	Annelida	*Capitella teleta*	Cleavage: in a single D-quadrant cell<br>Early gastrula: outside the blastopore in vegetal micromeres<br>Late gastrula: anterior surface cells encircling the site of stomodeum formation<br>Larvae: in subsurface oral	Involved in mouth formation and gut formation<br>Regulation of ectoderm differentiation	Boyle, Michael J., Emi Yamaguchi, and Elaine C. Seaver. "Molecular conservation of metazoan gut formation: evidence from expression of endomesoderm genes in Capitella teleta (Annelida)." EvoDevo 5.1 (2014): 1-19.

			ectoderm sur- rounding the buccal tube		
foxB	Mollusca	*Crepidula fornicata*	Cleavage: in each of the four quadrants, prominently to regions adjacent to the nuclei of the macromeres<br>Epiboly: in vegetal macromeres and in the progeny of the animal micromeres<br>Gastrulation: cells located around the blastopore<br>Elongation: mesenchyme and developing head	Ectomesoderm formation<br>Epithelial-mesenchymal transition at the blastopore lip	S
foxC	Phoronida	*Phoronopsis harmeri*	Blastula: posterior mesoderm<br>Late gastrula: posterior mesoderm<br>Larvae: posterior mesoderm	Posterior mesoderm formation	Andrikou, Carmen, and Andreas Hejnol. "FGF signaling acts on different levels of mesoderm development within Spiralia." Development 148.10 (2021): dev196089.
	Annelida	*Owenia fusiformis*	Blastula: absent<br>Gastrula: absent<br>Elongation: most anterior-lateral mesodermal cells and in a few cells in the anterior dorsal side of the embryo<br>Larvae: posterior pharyngeal mesoderm.	Mesodermal marker	Martín-Durán, José M., et al. "The developmental basis for the recurrent evolution of deuterostomy and protostomy." Nature ecology & evolution 1.1 (2016): 1-10.
		Helobdella austinensis	Cleavage: absent<br>Organogenesis: In prostomium and lateral mesoderm. Later in the ventral muscle and in the proboscis	Foregut formation	Kwak, Hee-Jin, et al. "Temporal and spatial expression of the Fox gene family in the Leech Helobdella austinensis." Journal of Experimental Zoology Part B: Molecular and Developmental Evolution 330.6-7 (2018): 341-350.
		Capitella teleta	Trochophore: developing mantle cavities, a bilateral pair of mesodermal cells posterior to the prototroch and a number of ventral mesodermal cells adjacent to the mouth and oesophagus	Posterior mesoderm formation/somatic mesoderm formation<br>Part of the *foxC-foxF-foxL1-foxQ1* cluster for mesoderm formation in metazoa	Shimeld, Sebastian M., et al. "Clustered Fox genes in lophotrochozoans and the evolution of the bilaterian Fox gene cluster." Developmental biology 340.2 (2010): 234-248.
	Brachiopod	*Novocrania anomala*	Blastula: ventral anterior mesoderm<br>Gastrula: ventral anterior mesoderm, , spatially separate from the blastoporal opening<br>Elongation: ventral anterior mesoderm<br>Larvae: ventral anterior mesoderm	Mesodermal marker	Martín-Durán, José M., et al. "The developmental basis for the recurrent evolution of deuterostomy and protostomy." Nature ecology & evolution 1.1 (2016): 1-10.
	Platyhelminth	*Schmidtea mediterranea*	*(two paralogs; expression is overlapping)*<br>Adults: around the pharynx and in the pharynx	Not determined	Pascual-Carreras, E., Herrera-Úbeda, C., Rosselló, M., Coronel-Córdoba, P., Garcia-Fernàndez, J., Saló, E., & Adell, T. (2021). Analysis of Fox genes in Schmidtea mediterranea reveals new families and a conserved role of Smed-foxO in controlling cell death. Scientific reports, 11(1), 1-18.

	Mollusca	*Terebratalia transversa*	Gastrula: anterior of the archenteron wall and broadly in the adjacent anterior ectoderm. Elongation: anterior archenteron wall and anterior ectodermal in the form of two lateral bands that extend along the animal-vegetal axis. Larvae: ventral anterior and posterior mesoderm.	Mesoderm formation Evolutionary conserved role for patterning mesoderm at both the anterior and posterior extremities	Passamaneck, Yale J., Andreas Hejnol, and Mark Q. Martindale. "Mesodermal gene expression during the embryonic and larval development of the articulate brachiopod Terebratalia transversa." Evodevo 6.1 (2015): 1-21.
		Patella vulgata	Early trochophore: cells visible in the mantle cavities and cells lying adjacent and anterior to the mouth, under the ectoderm Late trochophore:: mantle cavities, mesodermal cells posterior to the prototroch and adjacent to the foot and anterior to the prototroch and adjacent to the oesophagus	Posterior mesoderm formation/somatic mesoderm formation Part of the *foxC-foxF-foxL1-foxQ1* cluster for mesoderm formation in metazoa	Shimeld, Sebastian M., et al. "Clustered Fox genes in lophotrochozoans and the evolution of the bilaterian Fox gene cluster." Developmental biology 340.2 (2010): 234-248.
foxD	Annelida	*Platynereis dumerilii*	Adult: ventral somatic muscle markers	Ventral mesoderm marker	Lauri, Antonella, et al. "Development of the annelid axochord: insights into notochord evolution." Science 345.6202 (2014): 1365-1368.
	Platyhelminthes	*Schmidtea mediterranea*	Adult: anterior muscle cells	Not determined	Pascual-Carreras, E., Herrera-Úbeda, C., Rosselló, M., Coronel-Córdoba, P., Garcia-Fernàndez, J., Saló, E., & Adell, T. (2021). Analysis of Fox genes in Schmidtea mediterranea reveals new families and a conserved role of Smed-foxO in controlling cell death. Scientific reports, 11(1), 1-18.
	Brachiopod	*Terebratalia transversa*	Gastrula: narrow band of cells at the border of the archenteron wall and roof in the radial gastrula and in ectodermal cells at the anterior of the animal Elongation: narrow band of cells at the border of the archenteron wall and roof in the radial gastrula and broad band of ventral ectodermal expression just anterior of the blastopore Larvae: two bands of the mesoderm in the mantle lobe which converge ventromedially in the pedicle lobe	Ventral mesoderm formation	Passamaneck, Yale J., Andreas Hejnol, and Mark Q. Martindale. "Mesodermal gene expression during the embryonic and larval development of the articulate brachiopod Terebratalia transversa." Evodevo 6.1 (2015): 1-21.
foxE			Not determined		
foxF	Phoronida	*Phoronopsis harmeri*	Blastula: absent Late gastrula: anterior mesoderm Larvae: anterior and posterior mesoderm	Anterior and posterior mesoderm formation	Andrikou, Carmen, and Andreas Hejnol. "FGF signaling acts on different levels of mesoderm development within Spiralia." Development 148.10 (2021): dev196089.
	Brachiopoda	*Novocrania anomala*	Blastula: ventral anterior mesoderm Gastrula: ventral anterior mesoderm, , spatially separate from the blastoporal opening	Mesodermal marker	Martín-Durán, José M., et al. "The developmental basis for the recurrent evolution of deuterostomy and protostomy." Nature ecology & evolution 1.1 (2016): 1-10.

			Elongation: ventral anterior mesoderm<br>Larvae: ventral anterior mesoderm		
	Annelida	*Owenia fusiformis*	Blastula and gastrula: most anterior-lateral mesodermal cells and in a few cells in the anterior dorsal side of the embryo<br>Larvae: posterior pharyngeal mesoderm	Mesodermal marker	Martín-Durán, José M., et al. "The developmental basis for the recurrent evolution of deuterostomy and protostomy." Nature ecology & evolution 1.1 (2016): 1-10.
	Annelida	*Capitella teleta*	Trochophore: bilateral expression pattern in the brain, foregut and posterior ventro-lateral mesoderm	Posterior mesoderm formation/visceral mesoderm formation<br>Part of the *foxC-foxF-foxL1-foxQ1* cluster for mesoderm formation in metazoa	Shimeld, Sebastian M., et al. "Clustered Fox genes in lophotrochozoans and the evolution of the bilaterian Fox gene cluster." Developmental biology 340.2 (2010): 234-248.
	Platyhelminthes	*Schmidtea mediterranea*	Adult: cells in the margin of the head and muscle cells in the lateral dorsal part of animal, between the pharynx and the margin of the organism	Not determined	Pascual-Carreras, E., Herrera-Úbeda, C., Rosselló, M., Coronel-Córdoba, P., Garcia-Fernàndez, J., Saló, E., & Adell, T. (2021). Analysis of Fox genes in Schmidtea mediterranea reveals new families and a conserved role of Smed-foxO in controlling cell death. Scientific reports, 11(1), 1-18.
	Brachiopoda	*Terebratalia transversa*	Gastrula: absent<br>Elongation: anterior of the archenteron wall<br>Larvae: mesoderm laterally and anteriorly flanking the endoderm in larval stages	Mesoderm formation<br>Larval endoderm formation	Passamaneck, Yale J., Andreas Hejnol, and Mark Q. Martindale. "Mesodermal gene expression during the embryonic and larval development of the articulate brachiopod Terebratalia transversa." Evodevo 6.1 (2015): 1-21.
	Mollusca	*Patella vulgata*	Trochophore: in two bilateral pairs of cells, one level with the mouth and one anterior to the prototroch and their descendants in the lateral endoderm. Later, in the developing foot field	Posterior mesoderm formation/visceral mesoderm formation<br>Part of the *foxC-foxF-foxL1-foxQ1* cluster for mesoderm formation in metazoa	Shimeld, Sebastian M., et al. "Clustered Fox genes in lophotrochozoans and the evolution of the bilaterian Fox gene cluster." Developmental biology 340.2 (2010): 234-248.
foxG	Platyhelminthes	*Schmidtea mediterranea*	Adult: in a subset of muscle and neuronal cells all along the DV margin, in the dorsal midline and in some scattered cells in the dorsal and ventral part.	Not determined	Pascual-Carreras, E., Herrera-Úbeda, C., Rosselló, M., Coronel-Córdoba, P., Garcia-Fernàndez, J., Saló, E., & Adell, T. (2021). Analysis of Fox genes in Schmidtea mediterranea reveals new families and a conserved role of Smed-foxO in controlling cell death. Scientific reports, 11(1), 1-18.
foxG	Brachiopoda	*Terebratalia transversa*	Gastrula: two dis tinct domains in the animal cap ectoderm of the early radial gastrula<br>Elongation: Two additional, more lateral expression domains<br>Larvae: region of the	Anterior patterning<br>Ciliary band formation	Santagata, Scott, et al. "Development of the larval anterior neurogenic domains of Terebratalia transversa (Brachiopoda) provides insights into the diversification of larval apical organs and the spiralian

			transverse ciliated band and later in a 'U'- shape domain that borders the anterior ventral ectoderm		nervous system." Evodevo 3.1 (2012): 1-21.
foxH	Annelida	*Owenia fusiformis*	<u>Blastula:</u> Uniquely in the D-quadrant organiser cell <u>Gastrula:</u> Mesoderm precursors	Organizing activity	Seudre, O., Carrillo-Baltodano, A. M., Liang, Y., & Martín-Durán, J. M. (2021). ERK1/2 is an ancestral organising signal in spiral cleavage. *bioRxiv*.
foxI			Not determined	Not determined	
foxJ1	Annelida	*Platynereis dumerilii*	<u>Trochophore:</u> Ampullary cells, crescent cells and prototroch <u>Adult:</u> Ciliated cells	Formation of the apical organ Formation of the cilia	Marlow, H., Tosches, M. A., Tomer, R., Steinmetz, P. R., Lauri, A., Larsson, T., & Arendt, D. (2014). Larval body patterning and apical organs are conserved in animal evolution. BMC biology, 12(1), 1-17. And Pascual-Carreras, E., Herrera-Úbeda, C., Rosselló, M., Coronel-Córdoba, P., Garcia-Fernàndez, J., Saló, E., & Adell, T. (2021). Analysis of Fox genes in Schmidtea mediterranea reveals new families and a conserved role of Smed-foxO in controlling cell death. Scientific reports, 11(1), 1-18.
foxJ2/3			Not determined		
foxL1	Annelida	*Owenia fusiformis*	<u>Blastula and gastrula:</u> anterior portion of the mesodermal bands <u>Larvae:</u> lateral–posterior mesoderm of the pharynx.	Mesodermal marker	Martín-Durán, José M., et al. "The developmental basis for the recurrent evolution of deuterostomy and protostomy." Nature ecology & evolution 1.1 (2016): 1-10.
	Mollusca	*Patella vulgata*	<u>Blastula</u>: 4 micromere cells, in each embryonic quadrant. <u>Trochophore:</u> bilateral pair of mesoderm cells adjacent to the mouth in Later, two small expression domains are visible on either side of the mouth, lying under the ectoderm and adjacent to the epithelium of the oesophagus	Formation of the mesoderm that lines the external surface of the anterior gut	Shimeld, Sebastian M., et al. "Clustered Fox genes in lophotrochozoans and the evolution of the bilaterian Fox gene cluster." Developmental biology 340.2 (2010): 234-248.
		Capitella teleta	<u>Trochophore:</u> surface cells on the posterior face of the mouth, bilateral pair of patches on the dorsal–posterior side of the foregut, small domain bordering the left and right lateral–anterior rims of the pharynx pad and at very low levels in the brain and pharynx regions	Formation of the mesoderm that lines the external surface of the anterior gut	Shimeld, Sebastian M., et al. "Clustered Fox genes in lophotrochozoans and the evolution of the bilaterian Fox gene cluster." Developmental biology 340.2 (2010): 234-248.
foxL2/3	Annelida	*Helobdella austinensis*	<u>Cleavage:</u> absent <u>Organogenesis</u>: exterior germinal plate and later in mesodermal muscle fiber	Mesoderm development during late embryonic stage	Kwak, Hee-Jin, et al. "Temporal and spatial expression of the Fox gene family in the Leech Helobdella austinensis." Journal of Experimental Zoology Part B: Molecular and Developmental Evolution 330.6-7 (2018): 341-350.
foxQ1	Annelida	*Capitella teleta*	<u>Trochophore:</u> oesophagus in a highly asymmetric pattern between left and right sides of the animal with higher	Anterior gut formation	Shimeld, Sebastian M., et al. "Clustered Fox genes in lophotrochozoans and the evolution of the bilaterian Fox

				levels of expression on the left side		gene cluster." Developmental biology 340.2 (2010): 234-248.
	foxQ2	Annelida	*Platynereis dumerilii*	Trochophore: Upper two thirds of the episphere (the apical plate)<br>Adult: in differentiated eye cells, some brain progenitors and in ventral nerve cords	Formation of the apical organ	Marlow, H., Tosches, M. A., Tomer, R., Steinmetz, P. R., Lauri, A., Larsson, T., & Arendt, D. (2014). Larval body patterning and apical organs are conserved in animal evolution. BMC biology, 12(1), 1-17. And Pascual-Carreras, E., Herrera-Úbeda, C., Rosselló, M., Coronel-Córdoba, P., Garcia-Fernàndez, J., Saló, E., & Adell, T. (2021). Analysis of Fox genes in Schmidtea mediterranea reveals new families and a conserved role of Smed-foxO in controlling cell death. Scientific reports, 11(1), 1-18.
		Brachiopoda	*Terebratalia transversa*	Gastrula: Asymmetric domain shifted toward the presumptive dorsal end of the anterior ectoderm more<br>Elongation: Subset of anterior dorsal ectodermal domain<br>Larvae: anterior dorsal ectoderm of the apical lobe of the larva as well as a few small dorsal and ventral spots of expression	Anterior patterning<br>Apical tuft formation<br>Apical organ formation	Santagata, Scott, et al. "Development of the larval anterior neurogenic domains of Terebratalia transversa (Brachiopoda) provides insights into the diversification of larval apical organs and the spiralian nervous system." Evodevo 3.1 (2012): 1-21.
		Nemertea	*Lineus ruber*	Blastula: absent<br>Gastrula: sbsent<br>Larvae: most anterior region of the cephalic discs and in the proboscis<br>Juvenile: anterior head, including the proboscis	Apical organ formation	Martín-Durán, J. M., Vellutini, B. C., & Hejnol, A. (2015). Evolution and development of the adelphophagic, intracapsular Schmidt's larva of the nemertean Lineus ruber. *Evodevo*, *6*(1), 1-18.
II	*foxK*	Platyhelminthes	*Schmidtea mediterranea*	*(three paralogs, expression is overlapping)*<br>Adult: ubiquitously and specifically in the CNS	Not determined	Pascual-Carreras, E., Herrera-Úbeda, C., Rosselló, M., Coronel-Córdoba, P., Garcia-Fernàndez, J., Saló, E., & Adell, T. (2021). Analysis of Fox genes in Schmidtea mediterranea reveals new families and a conserved role of Smed-foxO in controlling cell death. Scientific reports, 11(1), 1-18.
	foxM			Not determined	Not determined	
	foxN1/4			Not determined	Not determined	
	foxN2/3	Mollusca	*Crepidula fornicata*	Cleavage: faintly and diffusely in cells in each of the four quadrants<br>Epiboly: In the micromere progeny<br>Gastrulation: around the blastopore lip<br>Elongation: entire lip of the blastopore expresses, including the ectomesodermal cells, in scattered mesenchymal cells leaving the lip of the blastopore, in some progeny of 4d<br>Larvea: in the developing head	Epithelial-mesenchymal transition at the blastopore lip	Osborne, C. C., Perry, K. J., Shankland, M., & Henry, J. Q. (2018). Ectomesoderm and epithelial–mesenchymal transition-related genes in spiralian development. *Developmental Dynamics*, *247*(10), 1097-1120.

	Platyhelminthes	*Schmidtea mediterranea*	*(two paralogs, expression is overlapping)*<br>Adult: ubiquitously in the SNC	Not determined	Pascual-Carreras, E., Herrera-Úbeda, C., Rosselló, M., Coronel-Córdoba, P., Garcia-Fernàndez, J., Saló, E., & Adell, T. (2021). Analysis of Fox genes in Schmidtea mediterranea reveals new families and a conserved role of Smed-foxO in controlling cell death. Scientific reports, 11(1), 1-18.
foxO	Annelida	*Helobdella austinensis*	*(two paralogs - overlapping expression)*<br>Cleavage: Ubiquitous<br>Organogenesis- FoxO1: Teloblasts and germinal band	Involved in cell division process during cleavage and perform a variety of functions throughout segmentation and organogenesis	Kwak, Hee-Jin, et al. "Temporal and spatial expression of the Fox gene family in the Leech Helobdella austinensis." Journal of Experimental Zoology Part B: Molecular and Developmental Evolution 330.6-7 (2018): 341-350.
foxO	Mollusca	*Crepidula fornicata*	Cleavage: diffusely throughout the micromeres<br>Epiboly: cells around the lip of the blastopore<br>Gastrulation: cells around the lip of the blas- topore, including the ectomesodermal progeny of $3a^2$ and $3b^2$ and in the mesentoblast<br>Elongation: cells around the blastopore<br>Larvae: head	General role in morphogenetic processes	Osborne, C. C., Perry, K. J., Shankland, M., & Henry, J. Q. (2018). Ectomesoderm and epithelial–mesenchymal transition-related genes in spiralian development. *Developmental Dynamics*, *247*(10), 1097-1120.
foxO	Platyhelminthes	*Schmidtea mediterranea*	Adult: ubiquitously	Role in controlling cell death	Pascual-Carreras, E., Herrera-Úbeda, C., Rosselló, M., Coronel-Córdoba, P., Garcia-Fernàndez, J., Saló, E., & Adell, T. (2021). Analysis of Fox genes in Schmidtea mediterranea reveals new families and a conserved role of Smed-foxO in controlling cell death. Scientific reports, 11(1), 1-18.
foxP	Platyhelminthes	*Schmidtea mediterranea*	Adult: specific parenchymal cell, type, pigment cells	Not determined	Pascual-Carreras, E., Herrera-Úbeda, C., Rosselló, M., Coronel-Córdoba, P., Garcia-Fernàndez, J., Saló, E., & Adell, T. (2021). Analysis of Fox genes in Schmidtea mediterranea reveals new families and a conserved role of Smed-foxO in controlling cell death. Scientific reports, 11(1), 1-18.

Supplementary Table 2. Genomic characteristics of the Fox genes in *O. fusiformis*

transcript id	*Fox* class	CDS Size (bp)	Number of introns
OFUSG21527.1	*foxA*	1374	0
OFUSG09476.1	*foxB*	888	0
OFUSG09642.1	*foxAB-1*	1353	0
OFUSG09693.1	*foxAB-2*	1134	2
OFUSG27049.1	*foxC*	1572	0
OFUSG13716.1	*foxD*	1488	0
OFUSG04768.1	*foxF*	1410	1
OFUSG25241.1	*foxG*	1081	0
OFUSG04867.1	*foxH*	1005	0
OFUSG10613.1	*foxJ1*	1323	1
OFUSG23528.1	*foxJ2/3*	1466	10
Manually curated	*foxK*	1179	–
OFUSG05364.1	*foxL1*	2223	0
OFUSG23682.1	*foxL2/3*	872	2
OFUSG08383.1	*foxM*	2170	7
Manually curated	*foxN1/4*	1140	2
OFUSG21828.1	*foxN2/3*	1536	6
OFUSG01726.1	*foxO*	1779	2
OFUSG05701.1	*foxP*	2042	11
OFUSG23111.1	*foxQ1*	1086	0
OFUSG03081.1	*foxQ2-1*	1222	0
OFUSG09682.1	*foxQ2-2*	1269	0
OFUSG09683.1	*foxQ2-3*	1091	0
OFUSG03083.1	*foxQ2-4*	1182	0
OFUSG03082.1	*foxQ2-5*	1182	0
OFUSG03084.1	*foxQ2-6*	1296	1
OFUSG08948.1	*foxQ2-7*	816	0
OFUSG09684.1	*foxQ2-8*	675	0
OFUSG20993.1	*foxQ2-9*	1017	0
Manually curated	*foxQ2-10*	1260	-
OFUSG19661.1	*foxQ2-11*	1440	0
OFUSG26073.1	*foxT*	3267	8
OFUSG26541.1	Orphan-1	1512	2
Manually curated	Orphan-2	2313	4
OFUSG18708.1	Orphan-3	3372	6

Supplementary Table 3. Timing of sample collection in four spiralian species

Species	Developmental stage	Time point	Number of replicates	Source
C. teleta	oocyte	0 hpf[1]	2	Martin Duran Lab
	zygote	~ 0.5 hpf	2	
	2-cell	~ 1h45 mpf	2	
	4-cell	~ 2 hpf	2	
	8-cell	~ 4h10 mpf[2]	2	
	16-cell	~ 6h10 mpf	2	
	32-cell	~ 8h10 mpf	2	
	blastula	~ 10h25 mpf	2	
	gastrula	~ 30h45 mpf	2	
	st4	~ 2 dpf[3]	2	
	st5	~ 3 dpf	2	
	St7	~ 5 dpf	2	
C. gigas	oocyte	0 hpf	1	Wang Jun et al. 2012. doi: 10.1038/nature11413.
	2-cell	1h20 mpf	1	
	4-cell	1h32 mpf	1	
	early morula	2h25 mpf	1	
	morula	3.5 hpf	1	
	blastula	4.5 hpf	1	
	rotary movement	5.5 hpf	1	
	free swimming	6.5 hpf	1	
	early gastrula (e. gastrula)	7.5 hpf	1	
	gastrula	8.5 hpf	1	
	trocophore	9.5 hpf	5	
	early D-shape larva	15.5 hpf	2	
	D-shape larva (D.larva)	17.5 hpf	7	
	early U-shape larva	5dpf	2	
	U-shape larva (U.larva)	10dpf	6	
	Late U-shape larva	14dpf	2	
	pediveliger	18dpf	2	
	spat	22dpf	1	
	juvenile	215dpf	1	
M. yessoensis	2-8cell	6 hpf	1	

	Blastula	18 hpf	1	Wang S et al. 2017. doi: 10.1038/s41559-017-0120.
	gastrula	28 hpf	1	
	trocophore	47 hpf	1	
	D-shape larva	70 hpf	1	
	pediveliger	26dpf	1	
	juvenile	30dpf	1	
O. fusiformis	oocyte	0 hpf	2	Martin Duran Lab
	zygote	0.5 hpf	2	
	2-cell	1 hpf	2	
	4-cell	1.5hf	2	
	8-cell	2 hpf	2	
	16-cell	3 hpf	2	
	32-cell	4 hpf	2	
	blastula	5 hpf	2	
	gastrula	9 hpf	2	
	Elongation (elong)	13 hpf	2	
	Early larva (e. larva)	17 hpf	2	
	Mitraria Larva	27 hpf	2	
	Co mpetent larva (c. larva)	3 wpf	2	
	Juvenile	1 hpm4	1	

[1] hpf: hour post fertilisation

[2] mpf: minute post fertilisation

[3] dpf: day post fertilisation

[4] hpm: hour post metamorphosis

Supplementary Table 4. Stage specific RNA-seq data (in TPM) for the *foxQ2* paralogs in *C. teleta*

Gene	Transcript ID	oocyte_rep1	oocyte_rep2	zygote_rep1	zygote_rep2	2cells_rep1	2cells_rep2	4cells_rep1
foxQ2-1	CTELG00000004951.1	626.45	396.52	418.56	333.22	247.13	458.14	293.41
foxQ2-2	CTELG00000015946.1	24.48	44.50	173.64	153.10	73.82	74.11	49.52
foxQ2-3	CTELG00000017495.1	0.00	0.00	0.00	0.00	1.26	0.00	0.00
foxQ2-4	CTELG00000018255.1	13,689.07	14,073.05	49,786.80	38,187.13	48,411.75	47,568.38	36,141.02
foxQ2-5	CTELG00000025237.1	634.26	1,116.09	295.36	219.44	204.91	104.59	190.35
foxQ2-6	CTELG00000026650.1	8.56	15.72	8.27	18.79	2.49	33.75	1.44

Gene	Transcript ID	4cells_rep2	8cells_rep1	8cells_rep2	16cells_rep1	16cells_rep2	32cells_rep1	32cells_rep2
foxQ2-1	CTELG00000004951.1	286.3	198.5	383.5	458.0	479.4	158.6	159.6
foxQ2-2	CTELG00000015946.1	64.2	63.9	49.6	312.5	530.3	957.8	1,511.1
foxQ2-3	CTELG00000017495.1	0.0	0.0	0.0	1.1	6.9	3.3	4.5
foxQ2-4	CTELG00000018255.1	37,925.3	19,499.8	22,487.0	8,013.4	7,744.2	3,201.0	2,591.6
foxQ2-5	CTELG00000025237.1	124.7	219.5	104.4	139.2	142.8	146.1	74.5
foxQ2-6	CTELG00000026650.1	1.2	15.2	19.6	12.2	47.4	104.9	117.2

Supplementary Table 5. Correspondence of foxQ2 genes in *C. gigas* and *M. yessoensis*

Species	Gene ID	Alignment ID	NCBI ID
C. gigas	*foxQ2-1*	FoxQ2	CGI_10003726
C. gigas	*foxQ2-2*	FoxQ2-D2	CGI_10002561
C. gigas	*foxQ2-3*	FoxQ2-D1	CGI_10006159
M. yessoensis	*foxQ2-1*	FoxQ2a	XM_021487993.1
M. yessoensis	*foxQ2-2*	FoxQ2c1	XM_021515362.1
M. yessoensis	*foxQ2-3*	FoxQ2b	XM_021492744.1
M. yessoensis	*foxQ2-4*	FoxQ2c2	XM_021504913.1
