# Supplementary figures and images for: The Fox Gene Repertoire in the Annelid Owenia fusiformis Reveals Multiple Expansions of the foxQ2 Class in Spiralia
Source: Genome Biol Evol. 2022 Sep 13;14(10):evac139. doi: 10.1093/gbe/evac139 (PMC9539403; doi:10.1093/gbe/evac139)

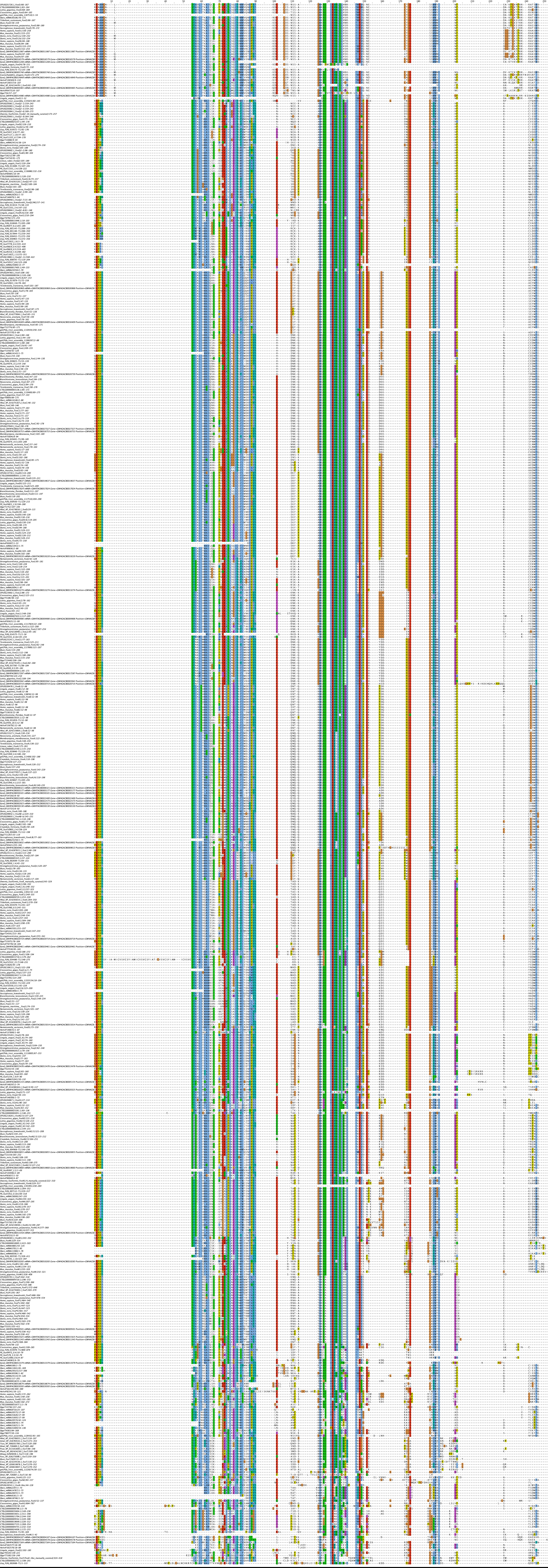

Supplement: evac139_Supplementary_Data [file evac139_supplementary_data.zip › FigureS1.png]

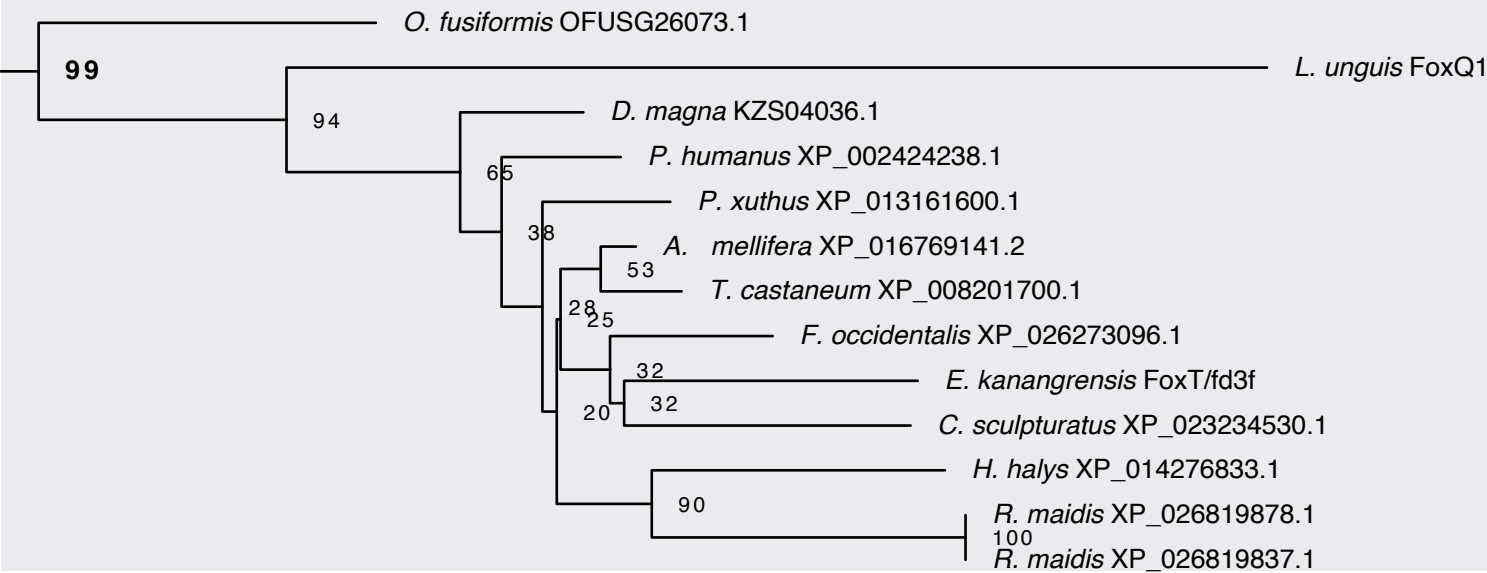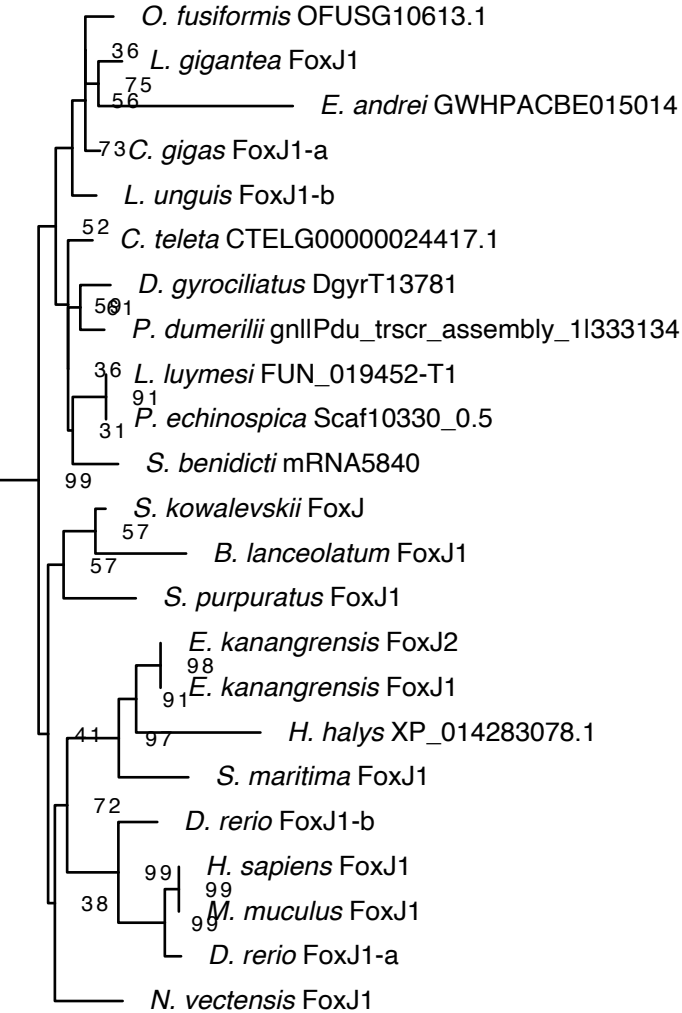

Supplement: evac139_Supplementary_Data [file evac139_supplementary_data.zip › FigureS4.pdf]

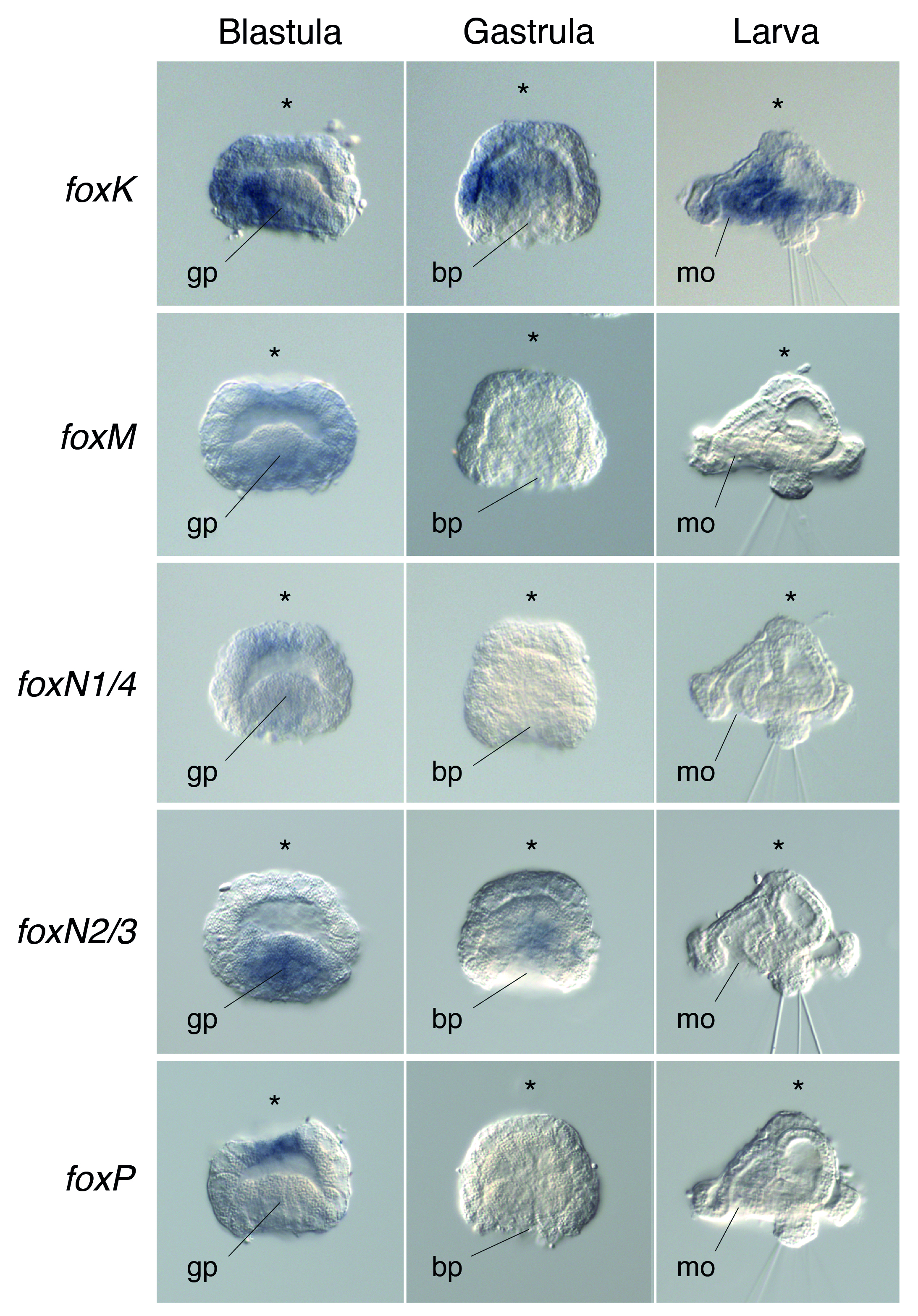

Supplement: evac139_Supplementary_Data [file evac139_supplementary_data.zip › FigureS5.jpg]

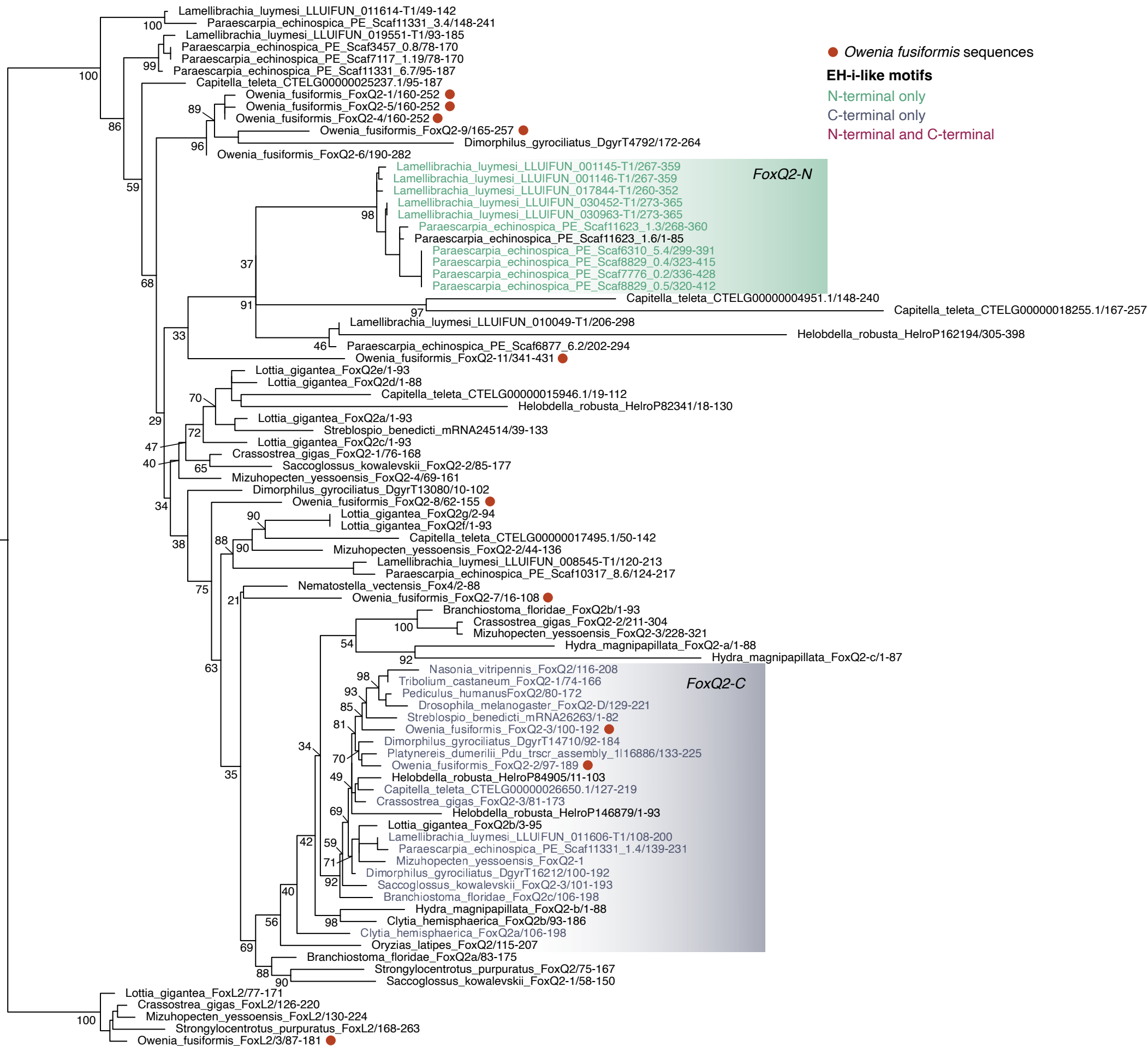

Supplement: evac139_Supplementary_Data [file evac139_supplementary_data.zip › FigureS7.pdf]
