# Supplementary material for: The Fox Gene Repertoire in the Annelid Owenia fusiformis Reveals Multiple Expansions of the foxQ2 Class in Spiralia
Source: Genome Biol Evol. 2022 Sep 13;14(10):evac139. doi: 10.1093/gbe/evac139 (PMC9539403; doi:10.1093/gbe/evac139)

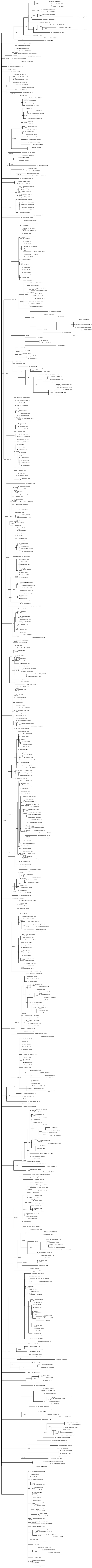

Supplement: evac139_Supplementary_Data [file FigureS3.pdf › FigureS3.pdf]
