# Supplementary material for: The Fox Gene Repertoire in the Annelid Owenia fusiformis Reveals Multiple Expansions of the foxQ2 Class in Spiralia
Source: Genome Biol Evol. 2022 Sep 13;14(10):evac139. doi: 10.1093/gbe/evac139 (PMC9539403; doi:10.1093/gbe/evac139)
Supplement: evac139_Supplementary_Data [file FigureS8.pdf › FigureS8.pdf]

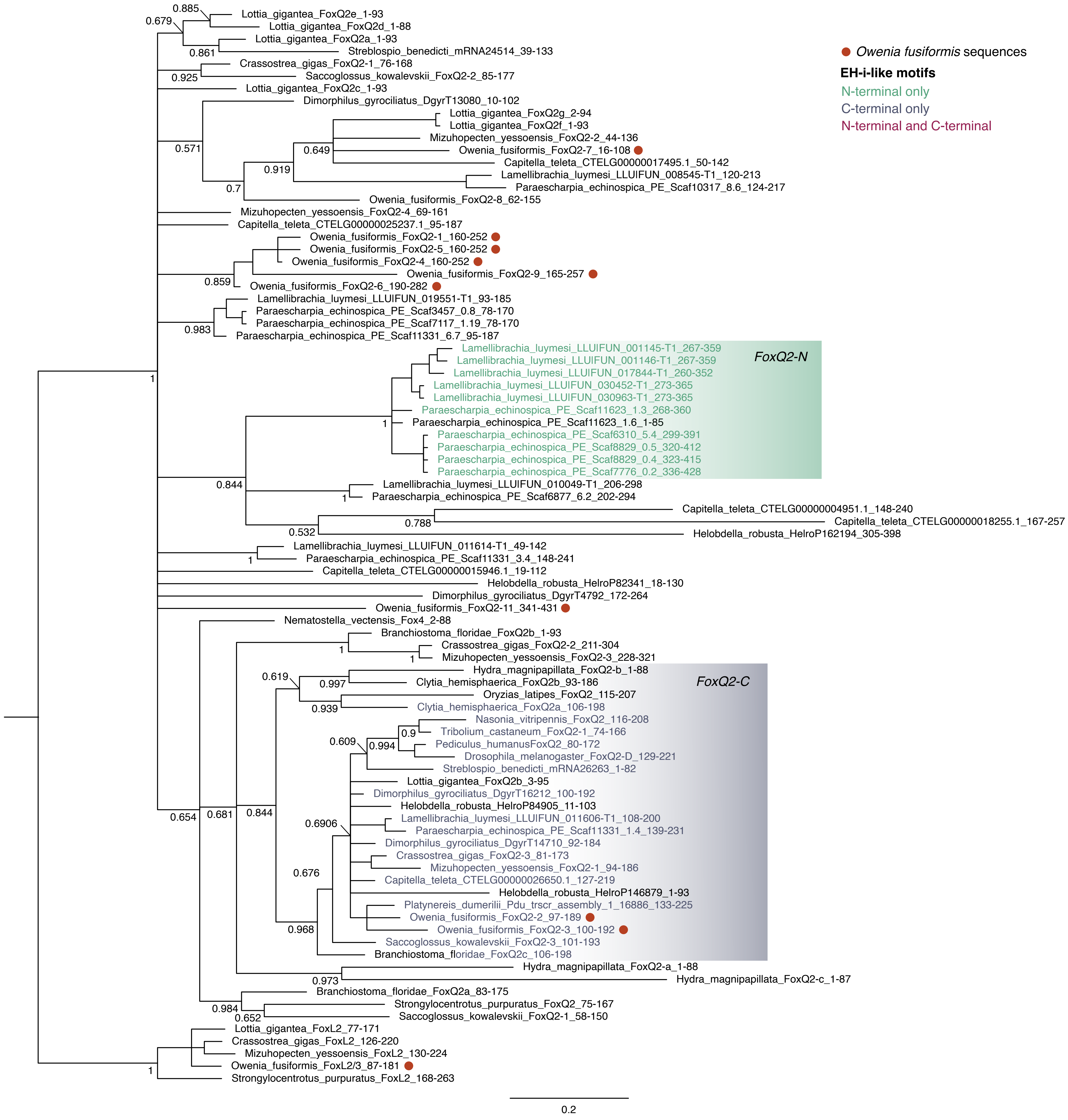

Owenia fusiformis sequences
EH-i-like motifs
N-terminal only
C-terminal only
N-terminal and C-terminal
FoxQ2-N
FoxQ2-C
Lottia_gigantea_FoxQ2e_1-93
Lottia_gigantea_FoxQ2d_1-88
Lottia_gigantea_FoxQ2a_1-93
Streblospio_benedicti_mRNA24514_39-133
Crassostrea_gigas_FoxQ2-1_76-168
Saccoglossus_kowalevskii_FoxQ2-2_85-177
Lottia_gigantea_FoxQ2c_1-93
Dimorphilus_gyrociliatus_DgyrT13080_10-102
Lottia_gigantea_FoxQ2g_2-94
Lottia_gigantea_FoxQ2f_1-93
Mizuhopecten_yessoensis_FoxQ2-2_44-136
Owenia_fusiformis_FoxQ2-7_16-108
Capitella_teleta_CTELG00000017495.1_50-142
Lamellibrachia_luymesi_LLUIFUN_008545-T1_120-213
Paraescharpia_echinospica_PE_Scaf10317_8.6_124-217
Owenia_fusiformis_FoxQ2-8_62-155
Mizuhopecten_yessoensis_FoxQ2-4_69-161
Capitella_teleta_CTELG00000025237.1_95-187
Owenia_fusiformis_FoxQ2-1_160-252
Owenia_fusiformis_FoxQ2-5_160-252
Owenia_fusiformis_FoxQ2-4_160-252
Owenia_fusiformis_FoxQ2-9_165-257
Owenia_fusiformis_FoxQ2-6_190-282
Lamellibrachia_luymesi_LLUIFUN_019551-T1_93-185
Paraescharpia_echinospica_PE_Scaf3457_0.8_78-170
Paraescharpia_echinospica_PE_Scaf7117_1.19_78-170
Paraescharpia_echinospica_PE_Scaf11331_6.7_95-187
Lamellibrachia_luymesi_LLUIFUN_001145-T1_267-359
Lamellibrachia_luymesi_LLUIFUN_001146-T1_267-359
Lamellibrachia_luymesi_LLUIFUN_017844-T1_260-352
Lamellibrachia_luymesi_LLUIFUN_030452-T1_273-365
Lamellibrachia_luymesi_LLUIFUN_030963-T1_273-365
Paraescharpia_echinospica_PE_Scaf11623_1.3_268-360
Paraescharpia_echinospica_PE_Scaf11623_1.6_1-85
Paraescharpia_echinospica_PE_Scaf6310_5.4_299-391
Paraescharpia_echinospica_PE_Scaf8829_0.5_320-412
Paraescharpia_echinospica_PE_Scaf8829_0.4_323-415
Paraescharpia_echinospica_PE_Scaf7776_0.2_336-428
Lamellibrachia_luymesi_LLUIFUN_010049-T1_206-298
Paraescharpia_echinospica_PE_Scaf6877_6.2_202-294
Capitella_teleta_CTELG00000004951.1_148-240
Capitella_teleta_CTELG00000018255.1_167-257
Helobdella_robusta_HelroP162194_305-398
Lamellibrachia_luymesi_LLUIFUN_011614-T1_49-142
Paraescharpia_echinospica_PE_Scaf11331_3.4_148-241
Capitella_teleta_CTELG00000015946.1_19-112
Helobdella_robusta_HelroP82341_18-130
Dimorphilus_gyrociliatus_DgyrT4792_172-264
Owenia_fusiformis_FoxQ2-11_341-431
Nematostella_vectensis_Fox4_2-88
Branchiostoma_floridae_FoxQ2b_1-93
Crassostrea_gigas_FoxQ2-2_211-304
Mizuhopecten_yessoensis_FoxQ2-3_228-321
Hydra_magnipapillata_FoxQ2-b_1-88
Clytia_hemisphaerica_FoxQ2b_93-186
Oryzias_latipes_FoxQ2_115-207
Clytia_hemisphaerica_FoxQ2a_106-198
Nasonia_vitripennis_FoxQ2_116-208
Tribolium_castaneum_FoxQ2-1_74-166
Pediculus_humanusFoxQ2_80-172
Drosophila_melanogaster_FoxQ2-D_129-221
Streblospio_benedicti_mRNA26263_1-82
Lottia_gigantea_FoxQ2b_3-95
Dimorphilus_gyrociliatus_DgyrT16212_100-192
Helobdella_robusta_HelroP84905_11-103
Lamellibrachia_luymesi_LLUIFUN_011606-T1_108-200
Paraescharpia_echinospica_PE_Scaf11331_1.4_139-231
Dimorphilus_gyrociliatus_DgyrT14710_92-184
Crassostrea_gigas_FoxQ2-3_81-173
Mizuhopecten_yessoensis_FoxQ2-1_94-186
Capitella_teleta_CTELG00000026650.1_127-219
Helobdella_robusta_HelroP146879_1-93
Platynereis_dumerilii_Pdu_trscr_assembly_1_16886_133-225
Owenia_fusiformis_FoxQ2-2_97-189
Owenia_fusiformis_FoxQ2-3_100-192
Saccoglossus_kowalevskii_FoxQ2-3_101-193
Branchiostoma_floridae_FoxQ2c_106-198
Hydra_magnipapillata_FoxQ2-a_1-88
Hydra_magnipapillata_FoxQ2-c_1-87
Branchiostoma_floridae_FoxQ2a_83-175
Strongylocentrotus_purpuratus_FoxQ2_75-167
Saccoglossus_kowalevskii_FoxQ2-1_58-150
Lottia_gigantea_FoxL2_77-171
Crassostrea_gigas_FoxL2_126-220
Mizuhopecten_yessoensis_FoxL2_130-224
Owenia_fusiformis_FoxL2/3_87-181
Strongylocentrotus_purpuratus_FoxL2_168-263
0.2
